# The Dogs of Tsenacomoco: Ancient DNA Reveals the Presence of Local Dogs at Jamestown Colony in the Early Seventeenth Century

**DOI:** 10.1017/aaq.2024.25

**Published:** 2024-05-22

**Authors:** Ariane E. Thomas, Matthew E. Hill, Leah Stricker, Michael Lavin, David Givens, Alida de Flamingh, Kelsey E. Witt, Ripan S. Malhi, Andrew Kitchen

**Affiliations:** 1 Department of Anthropology, University of Iowa, Iowa City, IA, USA; 2 Jamestown Rediscovery, Jamestown, VA, USA; 3 Center for Indigenous Science, Carl R. Woese Institute for Genomic Biology, University of Illinois, Urbana, IL, USA; 4 Department of Anthropology and Center for Indigenous Science, Carl R. Woese Institute for Genomic Biology, University of Illinois, Urbana, IL, USA; 5 Center for Human Genetics and Department of Genetics and Biochemistry, Clemson University, Clemson, SC, USA

**Keywords:** Dogs, ancient DNA, Native American, Indigenous people, Jamestown Colony, colonialization, perros, ADN antiguo, Nativo americano, comunidades indígenas, Colonia de Jamestown, colonización

## Abstract

Multiple studies have demonstrated that European colonization of the Americas led to the death of nearly all North American dog mitochondrial lineages and replacement with European ones sometime between AD 1492 and the present day. Historical records indicate that colonists imported dogs from Europe to North America, where they became objects of interest and exchange as early as the seventeenth century. However, it is not clear whether the earliest archaeological dogs recovered from colonial contexts were of European, Indigenous, or mixed descent. To clarify the ancestry of dogs from the Jamestown Colony, Virginia, we sequenced ancient mitochondrial DNA from six archaeological dogs from the period 1609–1617. Our analysis shows that the Jamestown dogs have maternal lineages most closely associated with those of ancient Indigenous dogs of North America. Furthermore, these maternal lineages cluster with dogs from Late Woodland, Hopewell, and Virginia Algonquian archaeological sites. Our recovery of Indigenous dog lineages from a European colonial site suggests a complex social history of dogs at the interface of Indigenous and European populations during the early colonial period.

## Resumen

Múltiples estudios han demostrado que la colonización europea de las Américas provocó la muerte y sustitución de casi todos los linajes mitocondriales de perros norteamericanos por linajes europeos en algún momento entre 1492 dC. y la actualidad. Los registros históricos indican que los colonos importaron perros de Europa a Norteamérica, donde los perros se convirtieron en objeto de interés e intercambio desde el siglo XVII dC. Sin embargo, no está claro si los primeros perros arqueológicos recuperados en contextos coloniales eran de ascendencia europea, indígena o mixta. Para aclarar la ascendencia de los perros de la colonia de Jamestown (Virginia), secuenciamos el ADN mitocondrial antiguo de seis perros arqueológicos del periodo 1609–1617 dC. Nuestro análisis muestra que los perros de Jamestown tienen linajes maternos más estrechamente asociados con los de perros antiguos indígenas de Norteamérica. Además, estos linajes maternos coinciden con los de los perros de los yacimientos arqueológicos de Late Woodland, Hopewell y Virginia Algonquian. Nuestra recuperación de linajes de perros indígenas en un yacimiento colonial europeo sugiere una compleja historia social de los perros en la interfaz de poblaciones indígenas y europeas durante el periodo colonial temprano.

European activity in North America during the seventeenth and eighteenth centuries established a dynamic and frequently tragic landscape in which Europeans disrupted the political, economic, and social authority of resident Native groups and later of enslaved African people brought to the region. Europeans used racial and social distinctions between themselves and Indigenous and African peoples to create cultural and legal barriers, intentionally increasing this separation (Campbell 1994). These boundaries had profound impacts on early Indigenous–European relationships and set the stage for future resentment and recurring conflicts. European social divisions and the quest to establish white authority and superiority in the Americas were widespread, likely affecting other closely associated species, especially dogs (Derr 2004; Foote 2022).

Europeans and Native Americans valued their dogs as important companion animals, and both cultures used dogs in similar ways (Quinlan 2021). Dogs (*Canis lupus familiaris*) were independently maintained by each society as faithful companions, workers, and symbols of identity. Indigenous peoples buried dogs as dedicatory offerings (Hill 2000), employed dogs as hunters (Roberts 2017) and pack animals (Eiselt 2022; Welker and Dunham 2019), and used dog fur for clothing and other woven goods (Lin et al. 2023). European colonial powers, including the Spanish, British, and French, regularly brought European dogs to their colonies in the Americas to help with daily tasks, such as catching pests, herding livestock, and hunting wild animals; to mark social status; and to attack Native Americans (Derr 2004). In later periods, White settlers used aggressive dogs to punish insubordination as a way of maintaining the institution of slavery (Parry and Yingling 2020).

Dogs, paradoxically, connected and created tension between European and Indigenous cultures that reflected the complicated and rapidly changing social landscapes during this time. White colonists took a keen interest in the local dogs they encountered and frequently recorded their physical and behavioral characteristics (e.g., Barbour 1986:111; Derr 2004; Hariot 1588:28). Colonial settlers often described Indigenous dogs as mongrels or curs to emphasize a perceived lack of apparent breeds and thus limited Indigenous control over these animals (e.g., Barton 1803; Kercsmar 2016). Indigenous peoples not only identified European dogs as a direct threat to their existence but also as a means of weakening the colonial enterprise of conquering North America (Ensminger 2022). Native communities tried to thwart colonial efforts to maintain the purity of European dog breeds by seeking European dogs that had certain desirable traits, despite attempts to prevent and even outlaw these actions.

In this article, we offer dogs as potential proxies to examine how Indigenous communities negotiated their relationship with rising colonial powers and changing material conditions while continuing to express their own lifeways. We use genetic analyses of archaeological dogs to explore the social entanglement intertwining colonization. Multiple studies demonstrate that a near-complete replacement of maternal Indigenous dog ancestry with European dog lineages occurred sometime between the late fifteenth century and the present day (Ní Leathlobhair et al. 2018; van Asch et al. 2013). Like other archaeological research that ignores the multifaceted nature of Indigenous presence and persistence within the ongoing context of colonialism (Panich 2013; Silliman 2005, 2010), the loss of Indigenous dogs is an underexplored issue of colonial impacts in the Americas. The timing and rate of Indigenous dog replacement have implications for understanding the ecological and cultural changes to Native American lifeways resulting from the influx of European dogs.

We focus on a small aspect of this complex colonial process by investigating the ancestry of canids from Jamestown colony, established in Tsenacomoco, the Algonquian name for the Powhatan chiefdom in the tidewater areas of the Chesapeake Bay that would later become the Commonwealth of Virginia. As the first permanent English colony in North America, it represents one of the earliest sites of long-term and continuous interactions between British settlers and Tsenacomocoans. Identifying whether canid bones at Jamestown are dogs and estimating the proportion of dog ancestry deriving from the Americas or Europe provide insight into European and Indigenous management of their dogs. Dogs with ancestry predominantly from Europe suggests that either British, Powhatan, or both groups kept their dogs from interacting with each other to maintain specific behaviors or observable phenotypes important to that group. In contrast, a high proportion of Indigenous dog ancestry suggests a more complex engagement between the British and Powhatan peoples at Jamestown and less emphasis on maintaining the separation between dogs and their association with settlers.

Jamestown is one of the few early European colonial sites with a high frequency of canid remains and evidence of Indigenous influence, which makes it a suitable site for this study. Archaeological investigations at French and British sites dating to the early seventeenth century, such as Sylvester Manor, New York (MNI = 1; Sportman et al. 2007); Ferryland, Newfoundland (MNI = 1; Welker and Dunham 2019); Utopia Plantation, Virginia (MNI = 1; Kelso 1984); Pettus Plantation, Virginia (MNI = 4; Kelso 1984); and Bray Plantation, Virginia (MNI = 1; Kelso 1984), have recovered remains identified as dog. However, these sites generally date 50 to 90 years after Jamestown, and the number of recorded canids is relatively small, often only one individual per site. Importantly, most of these early colonial sites did not have a substantial Indigenous presence except for Sylvester Manor, where Native Americans worked as laborers.

Jamestown’s canid collection also differs from other contemporary assemblages because the collection is relatively large (NISP = 181; MNI = 16) and the canid remains were recovered from multiple archaeological contexts. The canids at Jamestown are smaller in body size compared to dogs from other colonial sites, such as those from Sylvester Manor and Ferryland, which are likely large English breeds (Welker and Dunham 2019). Finally, unlike most other colonial sites, documentary accounts and archaeological evidence suggest that dogs at Jamestown were intensively butchered and consumed (Brown 1890:392; Generall Assembly in Virgina 1907:423; Haile 1998:895, 913).

This study generates new data on the genetics of these canids to confirm their identity as dogs and applies phylogenetic approaches to evaluate how Jamestown dogs linked British settlers with the Powhatan chiefdom. Our results can speak to the broader, colonial social landscape of this region and the ways early European colonists depended on local Native communities for their very survival, especially during the initial settlement period (Horn 2018). Our research has three main aims:

1. Confirm the species identification for the Jamestown canid remains using ancient DNA;
2. Conduct phylogenetic analyses of mitochondrial DNA obtained from Jamestown dogs to determine whether these remains are more closely related to Indigenous or European dog matrilineages;
3. Use results from our zooarchaeological analysis of the Jamestown dog assemblage to better document the size and composition of the remains and better understand the natural and cultural formation history of the assemblage.

## Tsenacomocoans, Colonists, and Dogs

British colonists at Jamestown came from a society with a long history of living and working with dogs (Degerbøl 1961; Harcourt 1974). Paintings, illustrated manuscripts, and statuaries depict dogs of different sizes and shapes living across Great Britain since at least Roman times (Friedman 2016; Johnston 1979). Written accounts from the Middle Ages and onward describe dogs serving as companions, guard animals, hunters, haulers, herders of livestock, and killers of pests (e.g., Cockayne 2016; Holmes 2017; Smith 1999). Well before the establishment of Jamestown, English dogs were assigned breed names, such as greyhound, beagle, mastiff, or spaniel, based on their distinctive body sizes, shapes, and behaviors (Berners 1881 [1486]; Caius 1576).

Dogs played prominent roles in the European colonial expansion into the Americas. Beginning with Columbus’s second voyage in 1493, most English and Spanish expeditions transported mastiffs, bloodhounds, and water spaniels to serve as camp guards, hunting aids, personal companions, and weapons to attack local Indigenous populations (e.g., Ensminger 2022; Lutz 1998; Strachey 1625; Varner and Varner 1983). In fact, England’s chief intellectual promoter of colonization, Richard Hakluyt (1877 [1584]:163) recognized that among the critical supplies needed for a successful colonial voyage were “Greyhounds to kill deere, &c. Mastives to kill heavie beastes of ravyne and for nighte watches. Bloude houndes to recover hurte dere.”

The Algonquian-speaking groups of Tsenacomoco that the English encountered when they arrived in Virginia had relied on dogs (*attomois or attemous* [Strachey 1625:175, 181]) for millennia. Tsenacomocoans not only valued dogs for obvious practical purposes like hunting birds and deer, hauling goods, and as a source of raw material but also recognized their ritual or spiritual value (Albizuri et al. 2019; Rountree 1989; Sharpe et al. 2022; Speck 1928). Archaeologists have uncovered evidence of Indigenous people burying their dogs, sometimes with humans, throughout the Chesapeake area. One of the most notable examples is a dog cemetery at the Weyanoke Old Town site (44PG51) that contained more than 100 individual dog burials (Blick 2000; Kelderhouse et al. 2012). Dog burials are strong mortuary evidence for the important spiritual and social value communities ascribed to the animals that extended beyond their utilitarian purpose in life.

On the colonists’ arrival to the Americas, dogs were a key point of interaction and sometimes disagreement among the Europeans and local Native communities. Spanish, French, and English colonists frequently reported subsisting on local (and occasionally European) dogs during times of scarcity or when hunting was not possible (e.g., Derr 2004; Haile 1998; Kellogg and Taylor 1917; Lane 1906). The trade of dogs played a role in establishing relationships between Jamestown colonists and local Native Virginian groups (Derr 2004; Schwartz 1997; Smith 1812). In 1608, Captain John Smith and Captain Newport of Jamestown gifted one of King James I’s white greyhounds to Paramount chief (*mamanatowick*) Wahunsenacawh (Powhatan). According to Captain Smith, the gift was treated with great respect: “He fed it as if he fed himself” (Haile 1998:166).

The high value placed on European dogs was witnessed in the spring of 1613, after the Jamestown colonists kidnapped Wahunsenacawh’s daughter, Matoaka (Pocahontas). As restitution for his daughter’s kidnapping, Wahunsenacawh demanded that the English give him “two bone combs such as Captain Newport had given him (the wooden ones his own men can make); an hundred fishhooks, or if he could spare it, rather a fishing seine; and a cat, and *a dog*” (Haile 1998:836–837; emphasis added), among other material goods. Although the true extent of the dog trade between the colonists and Indigenous people in the Tidewater region is uncertain, by 1619 the First Virginia Assembly moved to outlaw it, proclaiming, “That no man do sell or give any of the greater howes to the Indians, or any English dog of quality, as a mastive, greyhound, blood hound, land or water spaniel, or any other dog or bitch whatsoever, of the English race” (Kingsbury 1933:170).

English-bred dogs also served Jamestown as camp sentries and weapons against Tsenacomocoans. The aggressiveness of English mastiffs toward Tsenacomocoans was so great that the colony’s minister, George Thorpe, chose to publicly kill one of the colony’s mastiffs to placate Powhatan chief Opechancanough. However, after Tsenacomocoans attacked the colony, the English used dogs to systematically attack nearby Indigenous villages “by pursuing and chasing them with our horses, blood-Hounds to draw after them, and Mastiues [mastiffs] to teare them” (Kingsbury 1933:557).

## Jamestown (44JC001)

Located along the tidal coast of Chesapeake Bay, Virginia, Jamestown Island is a complex site with multiple occupations located on the northern bank of the James River (Kelso 2006). Earlier European attempts to establish an enduring presence in the region had failed (Bernhard 1992:600–601), likely because of similar hardships and dangerous conditions experienced by the founders of Jamestown. Within the first few days of arrival, Tsenacomocoans ambushed the colonists, and the English constructed James Fort, a triangular fortification with three bulwarks at each corner (Haile 1998:94) to ward off future attacks. By the end of the first year, approximately two-thirds of the original settlers had perished, succumbing to disease, malnutrition, or violence (Kupperman 2009). Subsequent voyages from England brought hundreds of additional colonists, but many died or left the colony soon after arrival. The colony was nearly abandoned in the spring of 1610 following a particularly harsh winter characterized by violence with neighboring tribes, drought, poor harvests, lack of supplies, and severe starvation known as the Starving Time. By 1620, the colony’s population stabilized at about 350 residents, with sustained population growth only occurring in later decades (Kupperman 2009).

The relationship between early Jamestown residents and the neighboring Native local communities was complex and fluctuated rapidly. There were periods when the two groups cooperated and participated in reciprocal exchange, while at other times there were deep cultural misunderstandings, mistrust, and violent confrontations (Horn 2006, 2018; Kupperman 2009). For the first few years, the colonists were able to establish alliances with at least some neighboring Tsenacomocoan groups, who willingly supplied food, labor, and other essentials to the colony in return for copper, other trade items, and promises the English would help defend Tsenacomoco from surrounding hostile groups (Fausz 1990; Quitt 1995).

Times of disagreement and violence arose when Tsenacomocoans recognized the growing permanency of Jamestown and were subject to increasing mistreatment and unreasonable demands made by the English. The Virginia Tidewater region experienced severe drought from 1606 to 1612, which limited agricultural productivity and made supplying the English with resources a severe burden for Tsenacomocoans (Blanton 2000; Stahle et al. 1998). In the summer of 1609, most nations in Tsenacomoco became hostile to the English and refused to trade with them. The colonists often responded by attacking Powhatan villages and stealing their harvests and supplies (Fausz 1990; Horn 2006, 2018). The Starving Time winter was a period of open warfare in and around Jamestown. Wahunsenacawh’s forces and their allies laid siege to Jamestown for six months between November 1609 and May 1610, trapping most of the colonists inside the fort (Bernhard 1992; Fausz 1990:26).

Contributing to the dynamic nature of this relationship was the lack of unified leadership directing interactions between Powhatans and the English. Wahunsenacawh held great influence over 30 communities in and around the Chesapeake Bay at the founding of Jamestown, but these populations were not politically or socially homogeneous (Kupperman 2009). Many groups established their own agendas for interactions with the colony (Gleach 2000). Similarly, the leadership of Jamestown Colony was not an entirely cohesive group and had different approaches in dealing with local Native communities (e.g., Haile 1998). Both sides quickly recognized that maintaining peaceful relations was becoming untenable because they both wanted to occupy the same physical spaces.

The colony became more self-sufficient economically after establishing a political alliance with Wahunsenacawh by 1614 (Horn 2006, 2018), but this marked the beginning of deteriorating relations with the Tsenacomocoans. As the colony’s population grew, more settlements were established along the James River, and coexistence between the Europeans and local communities became increasingly strained, primarily due to the English settling on prime agricultural lands, the introduction and spread of European diseases, drought, and English missionary efforts (Rountree 1989). When the English Crown took over the colony two years after a particularly violent attack in 1622, official colonial policy focused on expansion and political and social domination of local Tsenacomocoans and other Indigenous territories farther from James River (Horn 2018). By the mid-1600s, the English had established colonial rule over much of the region, and the Powhatan chiefdom faced continued loss of their territories, population decline, group consolidation, and subjugation to English (and later US) laws and policy (Rountree 1989).

## Materials and Methods

**Figure 1.**
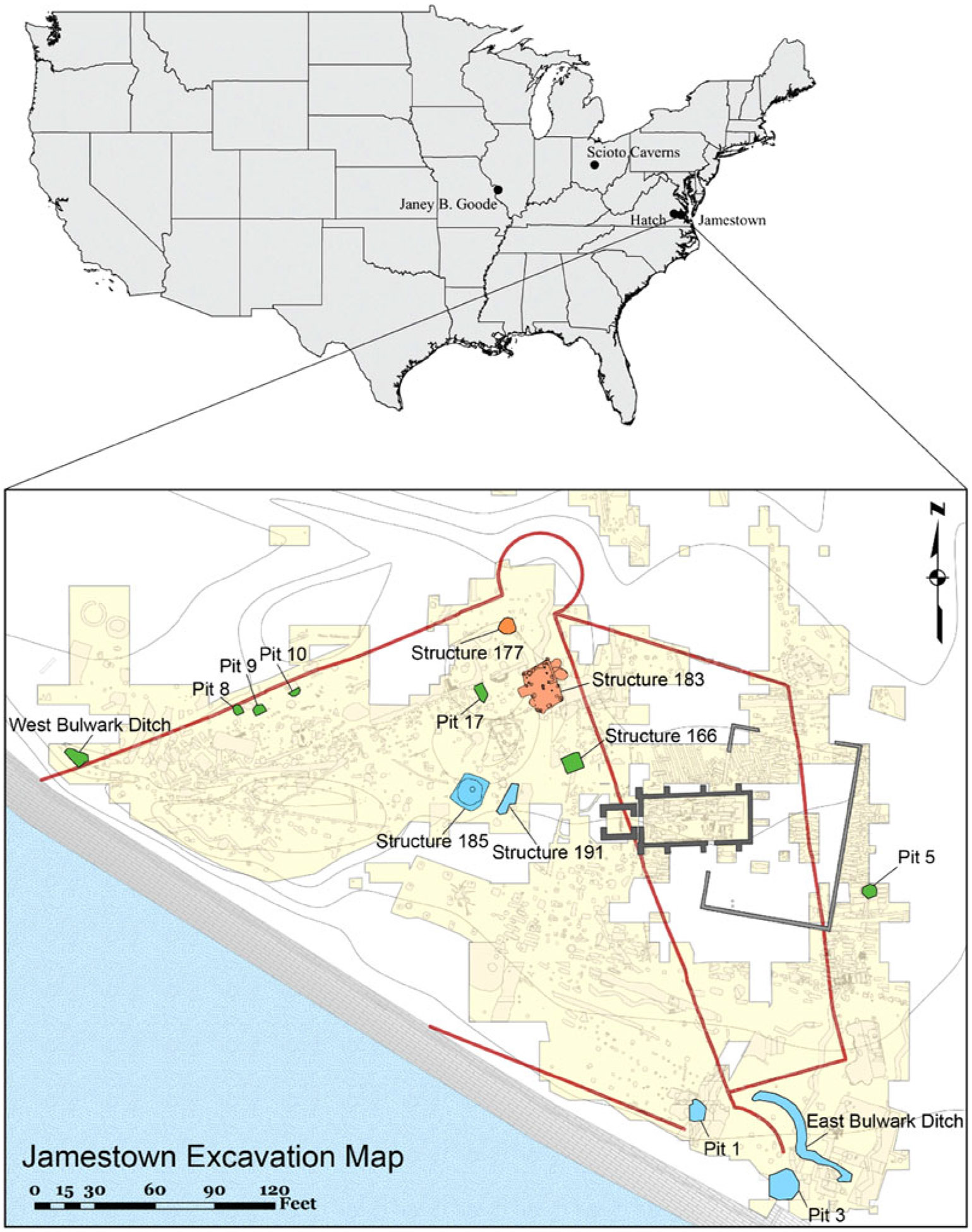
The upper panel shows the location of Jamestown and other archaeological sites (Scioto Caverns, Hatch/Weyanoke Old Town, and Janey B. Goode) that share genetic similarity with Jamestown dogs. The lower panel depicts the archaeological features producing dogs remains used in this study. Early settlement (AD 1607–1610) features are shown in green, Starving Time (AD 1609/1610) features are shown in blue, and post-Starving Time (AD 1610–1617) features are shown in orange. Structure 145 (Confederate Fort Pocahontas) is not shown on the map (base map courtesy of Jamestown Rediscovery Foundation, Preservation Virginia).

The Jamestown Rediscovery Archaeological Project excavated the canid remains used in this study. Since its inception in 1994, Jamestown Rediscovery’s archaeological team has excavated more than an acre within and around the triangular-shaped boundaries of the original James Fort, depicted as a red line in the second panel of Figure 1 (Kelso 2006). The presence of bulwarks (cannon emplacements) in each corner of the palisades and the recovery of arms and armor dating to the post-medieval period are evidence that this was a fortified area in the early seventeenth century. Further excavations recovered and identified the remains of a storehouse, kitchens, burials, soldier’s pits, barracks, and a space used for metallurgical testing and glassmaking. Archaeological finds representing the arrival of women and children, the explosion of tobacco as a commercial success, and materials reflecting the often-tense relationship between local Tsenacomocoans and the English highlight the nature of life at Jamestown in the years between 1607 and 1624.

Among the millions of faunal bones recovered at Jamestown, 181 bones have been identified as canid, representing at least 16 individuals. These remains were recovered from 15 different contexts that date to the first years of settlement (1607–1609), the Starving Time (winter of 1609/1610), and the post-Starving Time (1610–1617). The 22 canid bone samples subjected to DNA analysis were collected from nine contexts (Table 1; Figure 1). All the samples were recovered from well-dated, mixed trash deposits that filled abandoned occupation spaces (i.e., Pits 5, 8, 9, 17), a former workshop (Structure 183), two wells (Structures 177 and 185), and the construction trench for the fort’s defense walls (i.e., East bulwark trench; see Supplemental Text 1). In addition, a single dog bone of indeterminate age was recovered from a Civil War–era feature at the site (Structure 145); however, this feature disturbed earlier deposits so the age and specific context of this specimen are uncertain. There is no archaeological or historical evidence of Jamestown residents intentionally burying canids.

### Zooarchaeological Analyses

We conducted a zooarchaeological analysis of the canid collection found at Jamestown, in which we documented the features of the collection, such as NISP and MNI, and recorded evidence of cutmarks, impacts, pathologies, and other skeletal anomalies. We then selected 22 canid remains for sequencing because they represented multiple time periods within the fort’s history; had quality preservation of bone or tooth root, which increases the probability of extracting endogenous DNA; and represented individuals with and without cutmarks. To better understand the size of the Jamestown canids, we also measured the 22 samples using standard osteometric dimensions (von den Driesch 1976). We calculated their estimated body mass using linear regression equations developed by Losey and colleagues (2015) from modern dogs.

### Ancient DNA Extractions

Sample processing, DNA extractions, and library preparation of the 22 canid samples were conducted at the Carl R. Woese Institute for Genomic Biology at the University of Illinois at Urbana-Champaign (UIUC; see Table 2). The samples were rinsed with a bleach solution and DNA-free molecular grade water before being dried with ultraviolet light for about 30 minutes. Approximately 0.2 g of tooth root was drilled into a powder and then underwent a digestion protocol of 4 ml of 0.5 M EDTA, 100 μl of 3.33 mg/mL proteinase K, and 300 μl of 10% N-lauryl sarcosine that was incubated for 20 hours at 37°C following the procedures described in Cui et alia (2013). After digestion, the samples were spun at 3,500 rpm for five minutes to concentrate the bone powder from the liquid supernatant. The liquid supernatant was then passed through the centrifugal filter at 3,000 rpm until it was concentrated to 250 μl. The remaining supernatant was transferred to a new 1.5 ml tube. Manufacturer’s instructions were followed for the NEBNext Ultra II DNA Library Prep Kit (for more information on the extraction and NEB library protocol, see de Flamingh et al. 2023). The process from centrifugation to extraction was repeated to eliminate additional inhibitors.

### Library Construction

Genomic libraries for each sample were created at the Malhi Molecular Anthropology Laboratory at UIUC. Taphonomic factors degrade ancient DNA into fragments short enough to complete genomic library protocols without further cleavage. However, these short fragments are uneven and may have molecular overhangs susceptible to deamination, leading to the misincorporation of nucleotides during downstream processes. To prevent deamination and misincorporation, an end-repair protocol was used to blunt and phosphorylate the DNA fragments. Uracil DNA glycosylase was added to excise uracil nitrogenous bases from the fragments to further reduce the potential of miscoding damaged DNA. Afterward, unique NEBNext Multiplex Oligos (Unique Dual Indexes) for Illumina were ligated to each of the genomic libraries followed by a bead-elution. The samples were brought to a different laboratory space for amplification and underwent a Qiagen MinElute cleanup protocol following the manufacturer’s instructions. DNA libraries were evaluated using an E-Gel Power Snap System (ThermoFisher). They were quantified and sequenced using 2 X 150 bp paired-end sequencing on the NovaSeq 6000 and the NovaSeq X Plus platform at the Roy J. Carver Biotechnology Center at the UIUC.

### DNA Analyses

Raw reads for the Jamestown samples and samples sequenced by Ní Leathlobhair and colleagues (2018), da Silva Coelho and colleagues (2021), and Moon and colleagues (2023) were run through the PALEOMIX v1.3.7 ancient DNA pipeline for adapter removal, mapping, filtering, and damage analysis (Schubert et al. 2014). We generated consensus sequences by mapping each set of DNA reads to a publicly available dog mitochondrial genome (GenBank accession: NC_002008.4; Kim et al. 1998) using ANGSD 0.937 (Korneliussen et al. 2014). ANGSD parameters included using the most common base at a site to make a call (-doFasta 2 -doCounts 1) and filtering out the following: the first five bases on the end of each read (-trim 5), any reads with a mapping quality score below 30 (-minMapQ 30) and a minimum base quality score below 20 (-minQ 20), and any site where the sequencing depth is below 5 (-setMinDepth 5). Only samples with average sequence coverage of 5× or higher were used in downstream analyses. We performed multiple sequence alignment using MUSCLE (Edgar 2004) followed by manual curation on the six Jamestown dog sequences combined with modern and ancient dogs, coyotes, wolves, and foxes (*N* = 891) from GenBank and the European Nucleotide Archive (see the Data Availability Statement). A maximum likelihood tree was constructed using RAxML v.8.2 (Stamatakis 2014) with 500 bootstraps (-N 500 -m GTRGAMMAI --HKY85).

Given recent evidence of interspecific gene flow between multiple members of genus *Canis*, including coyotes and wolves (Gopalakrishnan et al. 2018), and known hybridization among dogs, wolves, and coyotes, we used red fox (*Vulpes vulpes*) as an outgroup. A Bayesian phylogenetic tree was constructed using only ancient dogs recovered from archaeological sites in North America (*n* = 67) with BEAST v1.10.4 (Suchard et al. 2018). The BEAST parameters included a strict clock, informative tip dates, a fixed mutation rate of 1.0 × 10^−7^ substitutions/site/year (informed by exploratory analyses and Ní Leathlobhair et al. 2018), a constant demographic model, and an HKY substitution model including a gamma distribution and proportion of invariant sites to accommodate site-rate heterogeneity. Multiple MCMC chains were run for 250 million generations and inspected for convergence in Tracer v1.7 (Rambaut et al. 2018) for all ancient North American dog lineages.

## Results

### Zooarchaeology and Body Mass Estimates

**Figure 2.**
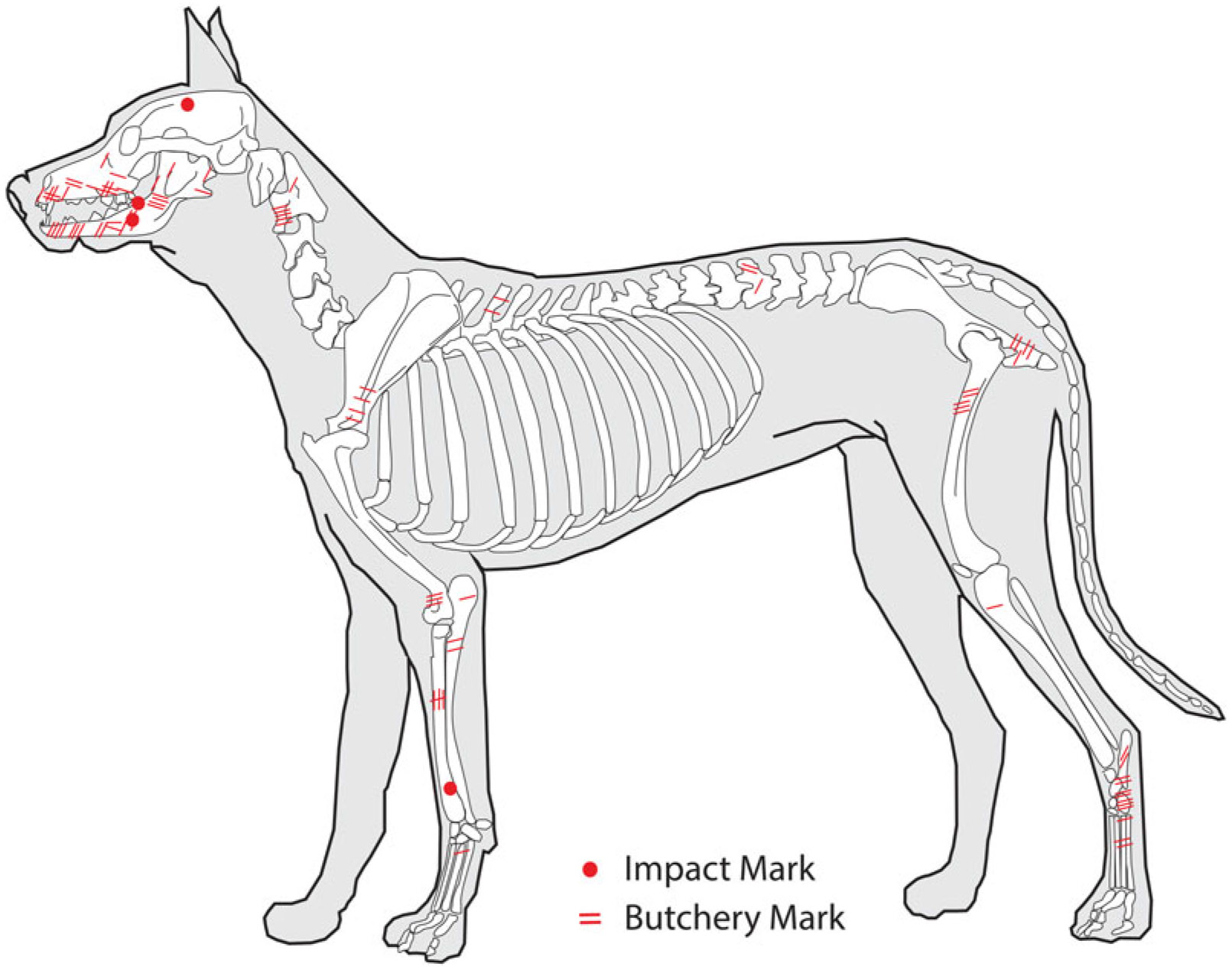
Composite map of butchery and impact marks across dog skeletons.

**Figure 3.**
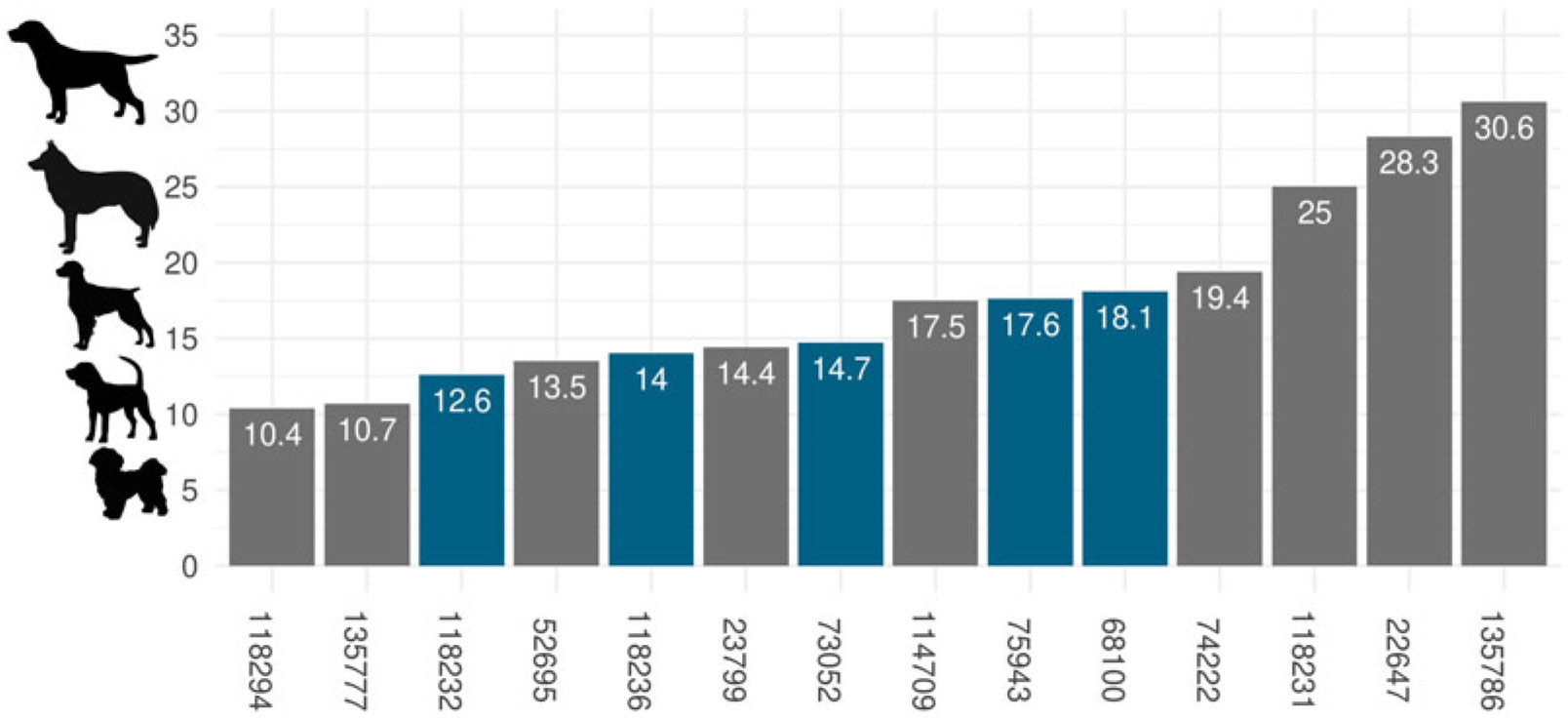
Estimated body masses of Jamestown dogs compared to modern dog breeds. Blue bars indicate samples with DNA greater than 5× for the mitochondrial genome, and gray bars indicate samples with lower-quality mitochondrial DNA (<5×). In ascending order from smallest to largest, Shih Tzus represent the smallest body masses, ranging from 4 to 7 kg, beagles are 9–14 kg, Brittany spaniels are 14–18 kg, Siberian huskies are 20–27 kg, and Labrador retrievers are 29–36 kg. Body masses are based on male dog averages provided by the American Kennel Club.

The Jamestown dog remains from all excavated features show evidence of human modification, including burning (NISP = 3), human-produced impacts (NISP = 4), and cutmarks (NISP = 48; Table 1, Figure 2). Of the 22 samples, 14 had measures that could be converted into body mass (refer to Supplemental Text 2:Table 2), which ranged from 10 kg to 31 kg (Figure 3). The largest specimens (#22647, #135786, and #118231) in the sample—dogs with body masses greater than 20 kg—are larger than all the other Jamestown dogs evaluated in this study. Most of the Jamestown dogs weighed between 10 and 19 kg. In modern dog breeds this range would be comparable in body size to a beagle, bull terrier, springer spaniel, or the standard schnauzer.

### Phylogenies

Twenty of the 22 Jamestown samples produced adequate libraries of the expected DNA fragment size for shotgun sequencing (Table 2). Five samples— #68100, #118294, #118236, #118231, and #118232—were sequenced twice to recover the maximum number of endogenous reads. All the samples produced DNA reads, but only six—#68100, #73052, #75943, #118232, #118236, and #135139—had an average dog mitogenome coverage greater than 5×.

**Figure 4.**
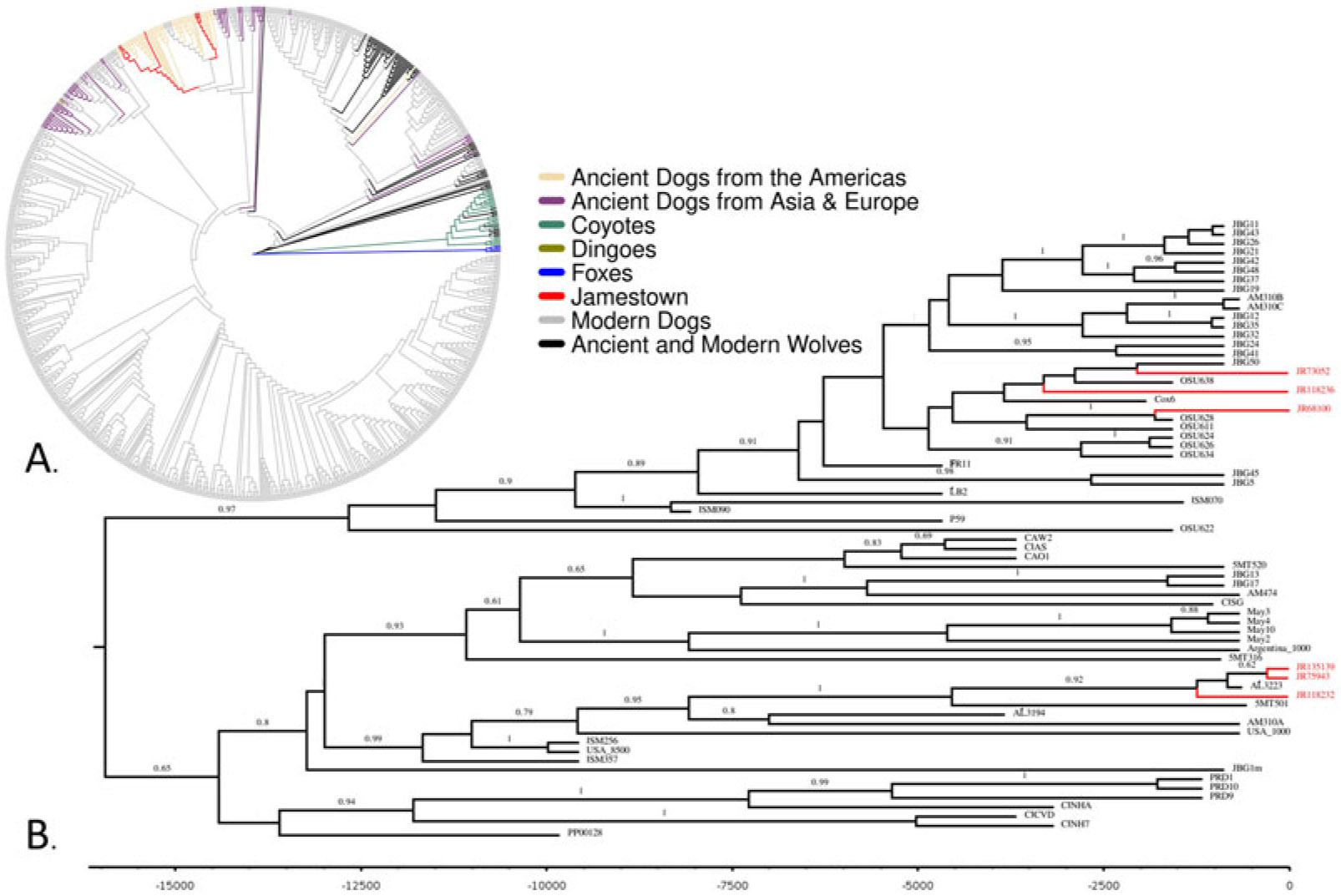
(A) Majority-rule maximum likelihood tree of ancient and modern canids (*N* = 891). *Vulpes vulpes* was used as an outgroup and is marked in blue. Ancient and modern wolves are colored black, modern coyotes are green, dingoes are dark yellow, ancient dogs from Asia and Europe are purple, ancient dogs from the Americas are tan, and modern dogs are gray; (B) Bayesian phylogeny of ancient North American canids. Y-axis is years before present. Posterior probabilities greater than 0.60 are labeled. Jamestown dogs are highlighted in red in both phylogenies.

The six Jamestown samples with highest DNA coverage—#68100, #118232, #118236, #73052, #75943, and #135139—clustered with sequences belonging to ancient North American dogs, all of which had been dated to before European contact in the maximum likelihood and Bayesian trees (Figure 4). The Jamestown dog sequences exhibit the closest affinity with precontact dogs from Scioto Caverns, Weyanoke Old Town, and Janey B. Goode sites (see Figure 1).

Scioto Caverns is a rockshelter locality in Franklin County, Ohio. Remains of at least 25 dogs were recovered in contexts containing human remains dating to the Hopewell period (Potter and Baby 1964). Avocational archaeologists collected the dog remains, so little information is available about the archaeological context of these dogs. However, the rockshelter is likely associated with the Holder-Wright earthworks (33FR4) complex within 100 m of the caverns (Shetrone 1924). Holder-Wright is a Middle Woodland period site (100 BC–AD 400) with three earthworks and five burial mounds.

Of the three sites, Weyanoke Old Town (44PG51), also known as the Hatch site, is the closest temporally and geographically to Jamestown dogs. It is approximately 60 km from Jamestown Colony and contains deposits dating from the Early Archaic through the Late Woodland period. The site was also occupied during the Late Woodland / contact period and is best known for its 112 dog burials and 34 human interments, making it one of the largest collections of archaeological canids ever recovered in North America (Blick 2000; Kelderhouse et al. 2012).

Finally, the Janey B. Goode (11S1232) site is located in southwestern Illinois and represents a moderately large collection of dog remains (NISP = 5,418) from burial and nonburial contexts (Kuehn 2014), as well as several dog coprolites (Witt et al. 2021). It was occupied from AD 650 to 1400, and there is evidence of dogs from multiple time points within this span. However, the dogs included in this study likely represent a narrower time frame, between AD 900 and 1050, during the Terminal Late Woodland period.

Fourteen samples did not yield sufficient DNA for downstream analyses because they had less than 5× coverage for the mitochondrial genome and were considered to have low quality DNA. However, we did attempt to generate sequences from these samples to estimate mitochondrial ancestry at the species level using BLAST and maximum likelihood trees. The maximum likelihood tree placed the low-quality Jamestown samples into clades containing ancient and modern dogs and outside wolf, fox, and coyote clades (see Supplemental Text 3:Figure 1). Of the low-quality sequences, seven—#52695, #118231, #118294, #135140, #135142, #135143, and #13577—clustered near the high-quality Jamestown samples in the ancient North American clade. The other seven (#22647, #23799, #74222, #114709, #135138, #135144, and #135786) were spread across clades containing modern dog breeds. Supplemental Text 2 summarizes the results pertaining to the samples with lower-quality endogenous DNA, and Supplemental Text 4 describes our evaluation of sequence authenticity.

## Discussion

These results show that at least six of the Jamestown dogs that we analyzed had unambiguous evidence of Indigenous North American ancestry. These dogs share mitogenomic similarities with Hopewellian, Mississippian, and Late Woodland period dogs from eastern North America. Our insights into the ancestry of these dogs are limited to their maternal ancestry because mitochondrial DNA is inherited unilaterally from mother to offspring. A focus of future study will be determining the remaining genetic ancestry of these dogs from nuclear DNA and continued work into increasing mitogenomic and nuclear DNA yield from the low-quality samples to confirm their genetic ancestries. It is possible that the six Jamestown dogs have full Indigenous dog ancestry, or they may be hybrids of early English male dogs and Indigenous female dogs. The latter outcome is plausible, and a similar example has been identified in Mutton, a Coast Salish woolly dog from the Pacific Northwest that died in 1859 (Lin et al. 2023). Mutton’s mitogenomic ancestry grouped with Indigenous dog lineages but also had about 16% European dog ancestry in its nuclear genome because of an European–Indigenous dog admixture event occurring 15.7–5.9 generations before Mutton’s birth (Lin et al. 2023). Our preliminary analysis of the low-quality samples suggests that all the remains used in our study are likely dogs, and seven of these specimens are potentially Indigenous dogs, whereas the remaining seven may have European ancestry. We caution against overinterpreting these low-coverage samples until more DNA can be acquired.

The identification of dogs with Indigenous ancestry at James Fort is not surprising, given the historical evidence for the Indigenous presence at Jamestown. There are numerous written accounts of Native Americans living and working in the colony (Kelso 2006), including Matoaka, who is arguably the most famous Native captive and later resident of Jamestown. In addition, the Spanish ambassador Pedro de Zúñiga reported to the king of Spain that at least 40 or 50 of the men at Jamestown had Native wives by 1612 (Brown 1890:572).

The recovery of large quantities of Indigenous artifacts within the same contexts as the dogs used in this study strongly supports the inference that Tsenacomocoans resided within James Fort in the early occupation of the colony. For example, Structure 185 contained several dog specimens and numerous Tsenacomocoan-made ceramic vessels, pipes, bone needles, nutting stones, and a burned reed mat (Kelso and Straube 2012). The feature was also filled with debris from bead production, including partially made beads and shaping stones (Kelso 2006). Excavations of Structure 166, a large rectangular earthen building used during the earliest occupation of the fort, yielded a nearly complete Indigenous-made pot associated with a cooking hearth, suggesting that Tsenacomocoans likely cooked within the structure (Kelso and Straube 2008:44–49). Powhatan society had pronounced gendered divisions of labor, in which women were involved in food preparation, farming, caregiving, and craftwork (Brown 1890; Rountree 1989). Thus, it is possible that Tsenacomocoan women were responsible for the presence of many of the Indigenous-produced artifacts at Jamestown Colony.

If the interpretation that Tsenacomocoans resided in Jamestown is correct, they may have introduced the Indigenous dogs to the fort. Dogs could have accompanied Tsenacomocoans as they moved in and out of the fort’s boundaries. The preservation of mostly Indigenous dog ancestry in our sample indicates that neither side prevented multiple local dogs from entering and staying at Jamestown. Tsenacomocoans permitted or at least overlooked the movement of their dogs into Jamestown or may have traded dogs with settlers. In either case, the colonists and Tsenacomocoans likely had little concern for possible interbreeding between these dogs and English dogs.

Indigenous dog ancestry in the fort is clear based on the results of this study, but is there evidence for European dogs at Jamestown? Ample documentary evidence (e.g., Archer 1607 quoted in Haile 1998:116; Sheppard and Ferrar 1919:4) suggests that colonists brought European dogs to Jamestown during their initial settlement and continued to import dogs throughout the colony’s tenure. However, the archaeological evidence for European dogs is more equivocal. Our genetic analysis of high-coverage samples did not identify dog ancestry derived from European breeds. However, seven low-quality sequences that grouped with modern dog lineages and were not associated with Indigenous dogs might represent European dog ancestry. However, the low quality of these genomes may have resulted in spurious associations, and other lines of evidence are necessary to demonstrate the presence of European dogs in the Jamestown assemblage.

In this case, our osteometric analyses might indicate that small Indigenous dogs and large European dogs are represented in the assemblage. The Jamestown colonists undoubtedly recognized that Tsenacomocoan dogs differed from European dogs in size and behavior. For example, Peter Wynne’s letter describes local dogs as “a Certeyne kind of Currs like our wariners hey dogges in England; and they keep them to hunt theyr land fowles, as Turkeys and such like, for they keep nothing tame about them” (Wynne 1608 quoted in Barbour 1969:246). Likewise, John Smith described local dogs, stating, “Their Dogges of that country are like their Wolves, and cannot barke but howle, and their wolves not much bigger than our English Foxes” (Smith 1612 quoted in Barbour 1969:349).

Although it is not clear what Wynne meant by “hey dog” (e.g., Rusek 2013), his use of the term “wariner” might indicate that he was drawing similarities to English dogs that hunted rabbits, foxes, badgers, and rats in and around warrens. If this is the case, then Indigenous dogs were probably recognized as small in body size, possibly the size of modern Scottish terrier, beagle, or basset hound breeds, which vary between 45 to 85 cm long and 25 to 42 cm at the shoulder and weigh 8–18 kg (American Kennel Club 2023). If Smith’s description is accurate, then the Native dogs were about the size of European red foxes (*Vulpes vulpes*) or roughly 70 cm long and 40 cm at the shoulder and between 4 and 18 kg in mass (Travaini and Delibes 1995). The English dogs brought to Jamestown, such as mastiffs, bloodhounds, and greyhounds, all have substantially larger body sizes. Welker and Dunham’s (2019) study of archaeological dogs from the colonial period on the East Coast supports the idea that European dogs were larger than Indigenous dogs found in the same area.

Most of the dogs in the Jamestown assemblage had body masses that ranged from 10 to 19 kg. Five of the six dogs (#68100, #73052, #75943, #118232, and #118236) produced body mass estimates between 12.6 and 18.1, which correspond to the smaller body sizes expected for Indigenous dogs. The largest specimens (#22647, #135786, and #118231) all had estimated body masses between 25 and 30 kg and may not represent Indigenous dogs. All three of these samples had low-quality DNA, and our preliminary phylogenetic analysis indicates that the smallest of the three specimens (#118231) may have Indigenous dog ancestry, whereas the two larger specimens (#22647 and #135786) possibly have European lineages.

### Consumption of Dogs at Jamestown

Dogs in this study were eaten by Jamestown residents during all three time periods between 1607 and 1617. These remains exhibited skeletal modifications consistent with human skinning, skeletal disarticulation, and meat removal. Butchered dog remains from Structure 185 unquestionably date to Jamestown’s Starving Time during the winter of 1609–1610, indicating that Indigenous dogs were eaten during this period of severe famine. However, butchered canid remains, including dogs identified as Indigenous, also come from early settlement (1607–1609) and post-Starving Time (1610–1617) contexts. The consumption of dogs suggests that Jamestown residents faced multiple periods of severe famine during the site’s early occupation and later periods. Although the consumption of dog flesh in modern Western societies is considered taboo, there is a long history of eating dogs during periods of stress in England and other parts of Europe (Harcourt 1974; Holmes 2017; Murphy 2001; Smith 1812). This behavior meant that the occupants of Jamestown acted like other early Spanish, English, and French colonists who consumed dog flesh in times of need (e.g., Derr 2004; Haile 1998; Kellogg 1917; Schwartz 1997).

## Conclusion

Six of the dogs from the Virginia Company period at Jamestown contained Indigenous ancestry and were consumed by the residents at Jamestown. The results of this study suggest complex forces at play before, during, and after the Starving Time that influenced the presence of these dogs in the fort and led the residents of Jamestown to consume dogs with Indigenous ancestry. Additional genetic sequencing, stable isotopes, and reconstructions of dog body size at Jamestown will shed more light on the complete ancestry, movements, and diets of dogs present during early European colonization and will help elucidate their role in a hybrid society that developed at Jamestown. Data from these dogs have potential implications for the commodification of dogs and their transient roles between Indigenous peoples and Europeans in the seventeenth century.

## Supplementary Material

Supplementary Text 3 Supplemental Text 3. Body Mass Estimates.

Supplementary Text 4 Supplemental Text 4. Sequence Authenticity.

Supplementary Text 1 Supplemental Text 1. Archaeological Context for Jamestown Samples for More Detailed Descriptions of the Features.

Supplementary Text 2 Supplemental Text 2. Low-Quality Data for Fourteen Jamestown Canids.

## Figures and Tables

**Table 1.** Count (NISP) of Canid Skeletal Elements by Context and Period.

Time Period/Context (deposit number)	NISP	Specimen Subject to DNA Analysis	Number of Burned Bones	Number of Bones with Impacts	Number with Cutmarks (percent of bones examined for butchery)
Early Settlement 1607–1610					
Pit 5: Possible Cellar (JR731A, B, D, F, M)	8	2, #22647, #23799	0	1	1 (25%)
Pit 8: Soldier’s Pit (JR1795D, E)	7	3, #135142, #135143, #135144	0	0	2 (50%)
Pit 9: Soldier’s Pit (JR1530A, B)	4	2, #52695, #135140	0	0	1 (50%)
Pit 10: Soldier’s Pit (JR1752B)	3	0	0	0	2 (67%)
Pit 17: Soldier’s Pit (JR2132H)	1	1, #135777	0	1	1 (100%)
Structure 166, Mud-and-Stud Soldier’s Pit (JT409L, M)	1	0	0	0	0
West Bulwark Trench (JR1425F)	1	0	0	0	1 (100%)
Starving Time 1609–1610					
Pit 1: Soldier and Borrow Pit (JR002V)	2	0	0	0	1 (50%)
Pit 3: Powder Magazine (JR124F, K)	3	0	0	0	1 (33%)
Structure 185: First Well (JR2718H, J, K, N, W)	76	9, #118230, #72592, #118236, #75943, #118231, #118232, #73052, #135786, #74222	3	1	16 (36%)
Structure 191: Cellar (JR3081B, C, F, G)	28	0	0	0	15 (60%)
East Bulwark Trench (JR081E, F, G, JR082W, X)	11	1, #114709	0	0	0
Post-Starving Time 1610–1617					
Structure 177: Second Well (JR2158H, N, P, U)	13	1, #135139	0	1	4 (36%)
Structure 183: Metalworking/Bakery Shop (JR2361A, C)	22	2, #135138, #68100	0	0	2 (67%)
Unknown Period					
Structure 145: Confederate Fort Pocahontas (JR1892D)	1	1, #118294	0	0	1 (100%)
Total	181	21	3	4	48 (42%)

**Table 2.** Mitochondrial and Nuclear DNA Coverage of Canid Samples.

Sample	Element	Sampled Location (for DNA)	Structure	Layer	mtDNA Depth of Coverage	mtDNA Breadth of Coverage (5×)
118294	Right mandible	Canine	145	JR1892D	4.14	35.79840
118230	Left maxilla	Maxilla superior to the fourth permanent premolar (unerupted)	185	JR2718J	Failed quality control at PCR stage (was not submitted for sequencing)	No DNA sequence
68100	Left maxilla	Fourth premolar	183	JR2361C	25.38	98.70870
118236	Right maxilla	Fourth premolar and a small amount from right first molar	185	JR2718W	66.79	99.04940
118231	Left maxilla	Fourth premolar	185	JR2718N	1.34	5.09954
118232	Left maxilla	Fourth premolar	185	JR2718N	5.32	71.93760^a^
73052	Left mandible	First molar	185	JR2718N	9.57	99.63530
75943	Right mandible	First molar	185	JR2718W	10.54	99.60540
135139	Right mandible	First molar	177	JR2158N	9.15	99.64730
114709	Left maxilla	Fourth premolar	East Bulwark	JR0082W	0.27	18.52100
135138	Right mandible	First molar	183	JR2361C	0.97	47.80890
135140	Right mandible	Canine	Pit 9	JR1530B	0.78	50.23610
135142	Left maxilla	Canine	Pit 8	JR1795D	0.67	43.76160
135143	Left mandible	Canine	Pit 8	JR1795D	0.43	32.31300
135144	Left mandible	Canine	Pit 8	JR1795D	1.16	62.56350
135786	Right mandible	Second and Third Premolar	185	JR2718N	1.54	71.16040
22647	Left mandible	First molar	Pit 5	JR0731F	1.04	61.92980
23799	Left mandible	First molar	Pit 5	JR0731D	0.19	16.66770
52695	Left mandible	First molar	Pit 9	JR1530B	1.19	63.41240
135777	Left maxilla	Second molar	Pit 17	JR2132H	0.68	43.56430
74222	Left mandible	First molar	185	JR2718N	2.59	89.96830
72592	Right Mandible	Inferior edge of the horizontal ramus	185	JR2718J	Failed quality control after fragment analyzer results (was not submitted for sequencing)	No DNA sequence

a Breadth of coverage done at 4×.

## Data Availability

Mitochondrial DNA sequences from the Jamestown dogs are available in GenBank accession numbers OR129837–OR129839 and SRA BioProject ID: PRJNA1068647, BioSample accessions SAMN39597810– SAMN39597829. Alignments and sample metadata are available on Github, https://github.com/thomasare/Dogs_of_Tsenacomoco_aDNA.
