# Supplementary material for: The Dogs of Tsenacomoco: Ancient DNA Reveals the Presence of Local Dogs at Jamestown Colony in the Early Seventeenth Century
Source: Am Antiq. Author manuscript; Available in PMC 2025 Aug 30. (PMC12395468; doi:10.1017/aaq.2024.25)
Supplement: Supplementary Text 3 — Supplemental Text 3. Body Mass Estimates. [file NIHMS2070856-supplement-Supplementary_Text_3.docx]

**Supplemental Text 3: Body Mass Estimates**

Supplemental materials for:

The Dogs of Tsenacomoco: Ancient DNA Reveals Presence of Local Dogs at Jamestown Colony in Early Seventeenth Century

**Authors**

Ariane Thomas*

Matthew E. Hill, Jr.

Leah Stricker

Michael Lavin

David Givens

Alida de Flamingh

Kelsey E. Witt

Ripan S. Malhi

Andrew Kitchen

*****Corresponding author, [ariane-thomas@uiowa.edu](mailto:ariane-thomas@uiowa.edu)

^§^These authors co-supervised this work.

**Contents**

Supplemental Table 1. Body Mass Estimations of Canids from Jamestown.

Supplemental Table 1. Body Mass Estimations of Canids from Jamestown.

| Sample Number | Structure | Layer | Measurement | Side | Measurement (cm) | Estimated body mass based on Losey *et al.* 2015 (kg) |
| --- | --- | --- | --- | --- | --- | --- |
| 118294 | 145 | JR1892D | VDDm12 | Right | 28.4 | 10.4 |
| 118230 | 185 | JR2718J | - | - | - | - |
| 68100 | 183 | JR2361C | VDDc18 breadth | Left | 10.2 | 18.1 |
| 118236 | 185 | JR2718W | VDDc18 breadth | Right | 9.4 | 14 |
| 118231 | 185 | JR2718N | VDDc18 breadth | Left | 11.3 | 25 |
| 118232 | 185 | JR2718N | VDDc20 breadth | Left | 13.5 | 12.6 |
| 73052 | 185 | JR2718N | VDDm2 | Left | 128.7 | 14.7 |
| 75943 | 185 | JR2718W | VDDm18 | Right | 53.8 | 17.6 |
| 135139 | 177 | JR2158N | - | - | - | - |
| 114709 | East Bulwark | JR0082W | VDDc18 breadth | Left | 10.1 | 17.5 |
| 135138 | 183 | JR2361C | - | - | - | - |
| 135140 | Pit 9 | JR1530B | - | - | - | - |
| 135142 | Pit 8 | JR1795D | - | - | - | - |
| 135143 | Pit 8 | JR1795D | - | - | - | - |
| 135144 | Pit 8 | JR1795D | - | - | - | - |
| 135786 | 185 | JR2718N | VDDm12 | Right | 40 | 30.6 |
| 22647 | Pit 5 | JR0731F | VDDm18 | Left | 65.8 | 28.3 |
| 23799 | Pit 5 | JR0731D | VDDm2 | Left | 127.7 | 14.4 |
| 52695 | Pit 9 | JR1530B | VDDm2 | Right | 124.9 | 13.5 |
| 135777 | Pit 17 | JR2132H | VDDc3 | Left | 140.8 | 10.7 |
| 74222 | 185 | JR2718N | VDDm18 | Left | 56 | 19.4 |
