# Supplementary material for: The Dogs of Tsenacomoco: Ancient DNA Reveals the Presence of Local Dogs at Jamestown Colony in the Early Seventeenth Century
Source: Am Antiq. Author manuscript; Available in PMC 2025 Aug 30. (PMC12395468; doi:10.1017/aaq.2024.25)

**Supplemental Text 4: Sequence Authenticity**

Supplemental materials for:

The Dogs of *Tsenacomoco*: Ancient DNA Reveals Presence of Local Dogs at Jamestown Colony in Early Seventeenth Century

**Authors**

Ariane Thomas*

Matthew E. Hill, Jr.

Leah Stricker

Michael Lavin

David Givens

Alida de Flamingh

Kelsey E. Witt

Ripan S. Malhi

Andrew Kitchen

*****Corresponding author, [ariane-thomas@uiowa.edu](mailto:ariane-thomas@uiowa.edu)

^§^These authors co-supervised this work.

**Contents**

Sequence Authenticity Description

[Supplemental Figure 1: mapDamage base frequencies for #118236.](#_Toc157785907)

[Supplemental Figure 2: mapDamage base frequencies for #118232.](#_Toc157785908)

[Supplemental Figure 3: mapDamage base frequencies for #68100.](#_Toc157785909)

[Supplemental Figure 4: mapDamage base frequencies for #73052.](#_Toc157785910)

[Supplemental Figure 5: mapDamage base frequencies for #75943.](#_Toc157785911)

[Supplemental Figure 6: mapDamage base frequencies for #135139.](#_Toc157785912)

[Supplemental Figure 7: Misincorporation for samples A) #118236 and B) #68100.](#_Toc157785913)

[Supplemental Figure 8: Misincorporation for samples A) #118232 and B) #73052.](#_Toc157785914)

[Supplemental Figure 9: Misincorporation for samples A) #75943 and B) #135139.](#_Toc157785915)

**Sequence Authenticity Description**

Work in the ancient DNA laboratory at UIUC is performed with strict measures in place to prevent DNA contamination. All equipment used in DNA extractions was exposed to UV light for at least 10 minutes prior to use. All lab surfaces were cleaned with a DNA degrading solution (bleach or Takara DNA-off^TM^) before and after any procedural steps in the protocol. A blank extraction and negative controls were processed with DNA extracted from the Jamestown dogs, including sequencing. Sample negatives for each of the four extractions sequenced with these samples had a mean coverages of the dog mitogenome that were at least one order of magnitude lower than lowest coverage sample analyzed and multiple orders of magnitude lower than the six samples used for phylogenetic analysis in the main manuscript. Lower mean coverages are consistent with little to no DNA contamination of the Jamestown samples.

We also performed bioinformatic analysis of the ancient DNA sequences to identify potential modern contamination. Ancient DNA is characteristically composed of highly fragmented short sequences with an elevated rate of nucleotide misincorporations above that of modern DNA. The sequences generated from the Jamestown *Canis* samples are highly fragmented and were <250 bp in length. The average read length for the samples was 125 bp for #68100, 124 bp for #118232, 128 bp for #118236, 125 bp for #73052, 130 bp for #75943, and 129 for #135139, which are typical for ancient samples. mapDamage v2.2.1 (Jónsson *et al.* 2013) was used to visualize C>T misincorporations on the 5’ end and G>A on the 3’ end of DNA sequence fragments. We found that the sequence fragments sequenced from the Jamestown *Canis* libraries had rates of nucleotide misincorporation consistent with ancient DNA (see supplemental material Figures 5-13). In sum, both sequence fragment size and condition analyses support the ancient origin of these mitogenome sequences.

The possibility DNA from other ancient dogs contaminated our samples analyzed in the same laboratory is unlikely given our strict measures to degrade all free DNA in the lab, and our analysis of the ancient mitogenomes does not support ancient contamination. If the sequences we generated were produced via contamination with DNA from a previous ancient sample, our phylogenetic analysis of the Jamestown sequences would have either placed them as closely related to or identical to existing ancient *Canis* sequences or as outliers with very long branches (due to the creation of a pseudo-recombinant sequence incorporating both authentic Jamestown dog and contaminated dog DNA fragments). Instead, our analysis produced a tree that placed the Jamestown *Canis* sequences with sequences from the Scioto Cavern, Weyanoke Old Town (also known as the Hatch site), and Janey B. Goode sites, and none of the high-quality Jamestown sequences had long terminal branches in relation to other ancient sequences. Importantly, the Janey B. Goode dogs (except sample JBG1m) and the Hatch dogs were sequenced at another ancient DNA facility, and all the samples from Janey B. Goode, Weyanoke Old Town, and Scioto Caverns were sequenced at least three years prior to our study. This makes it unlikely that the mitogenome sequences generated here result from contamination from previous dog samples sequenced at UIUC. Additionally, because no European dog lineages were recovered, it is also very unlikely that modern dogs contaminated our reads.

Supplemental Figure 1: mapDamage base frequencies for #118236.

.
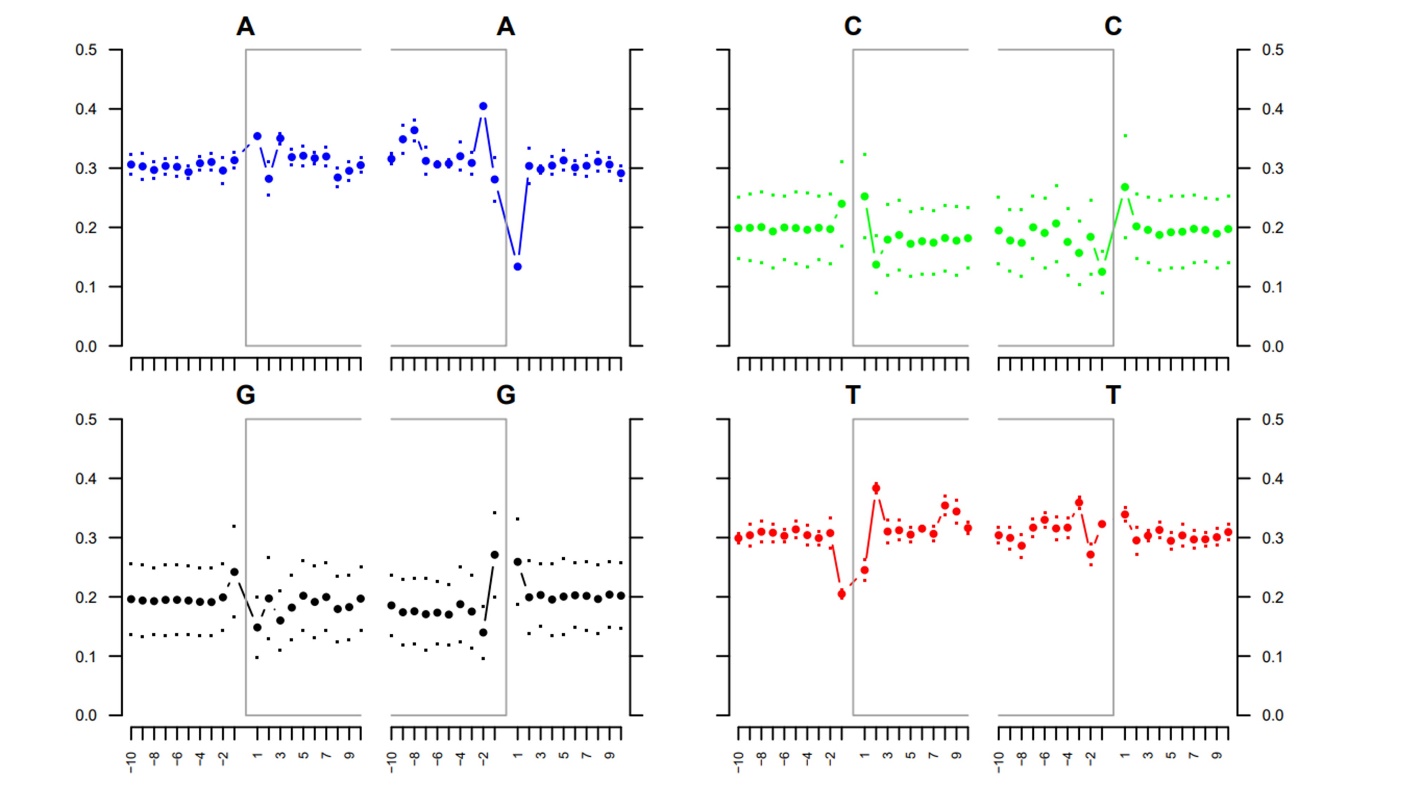


Supplemental Figure 2: mapDamage analysis base frequencies for #118232.


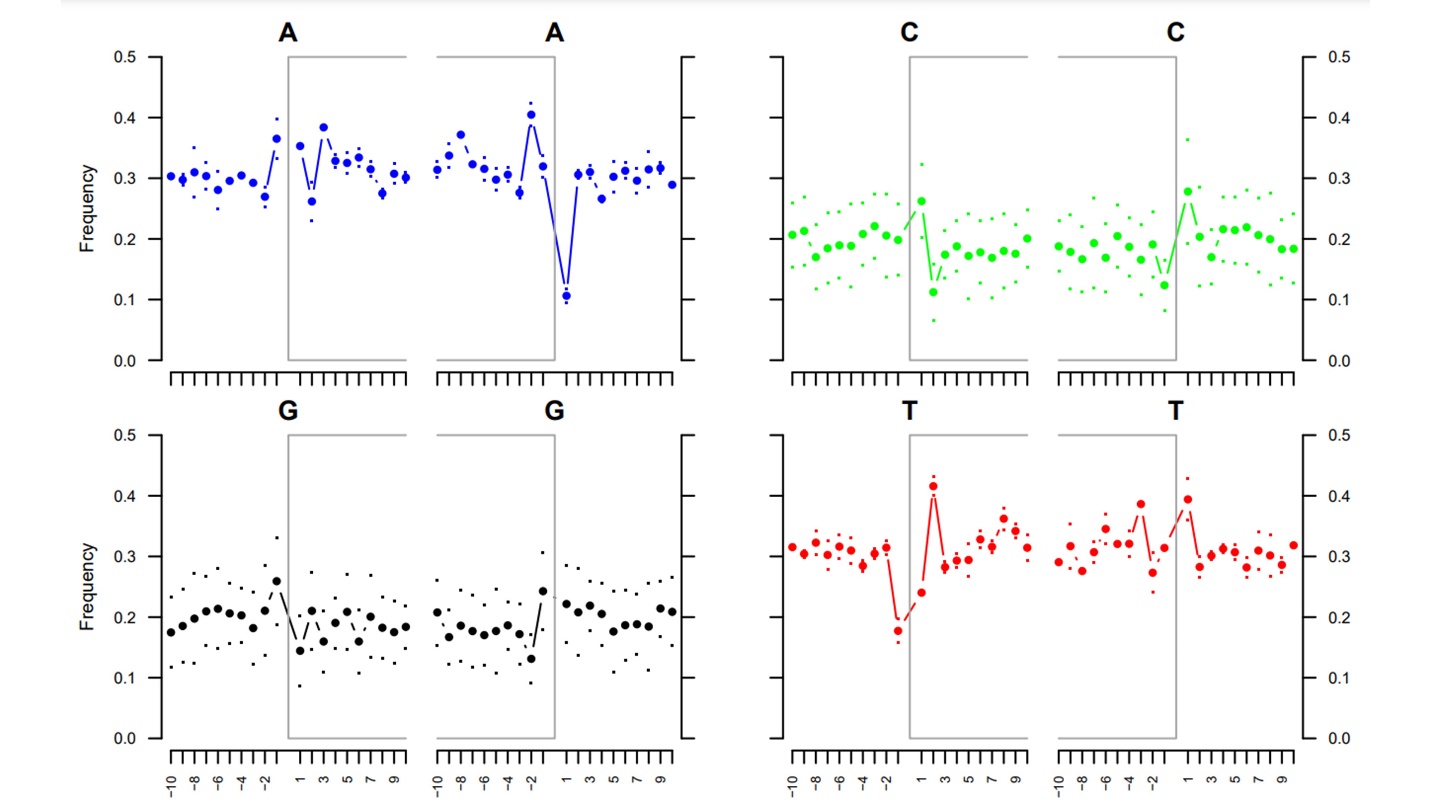


Supplemental Figure 3: mapDamage base frequencies for #68100.


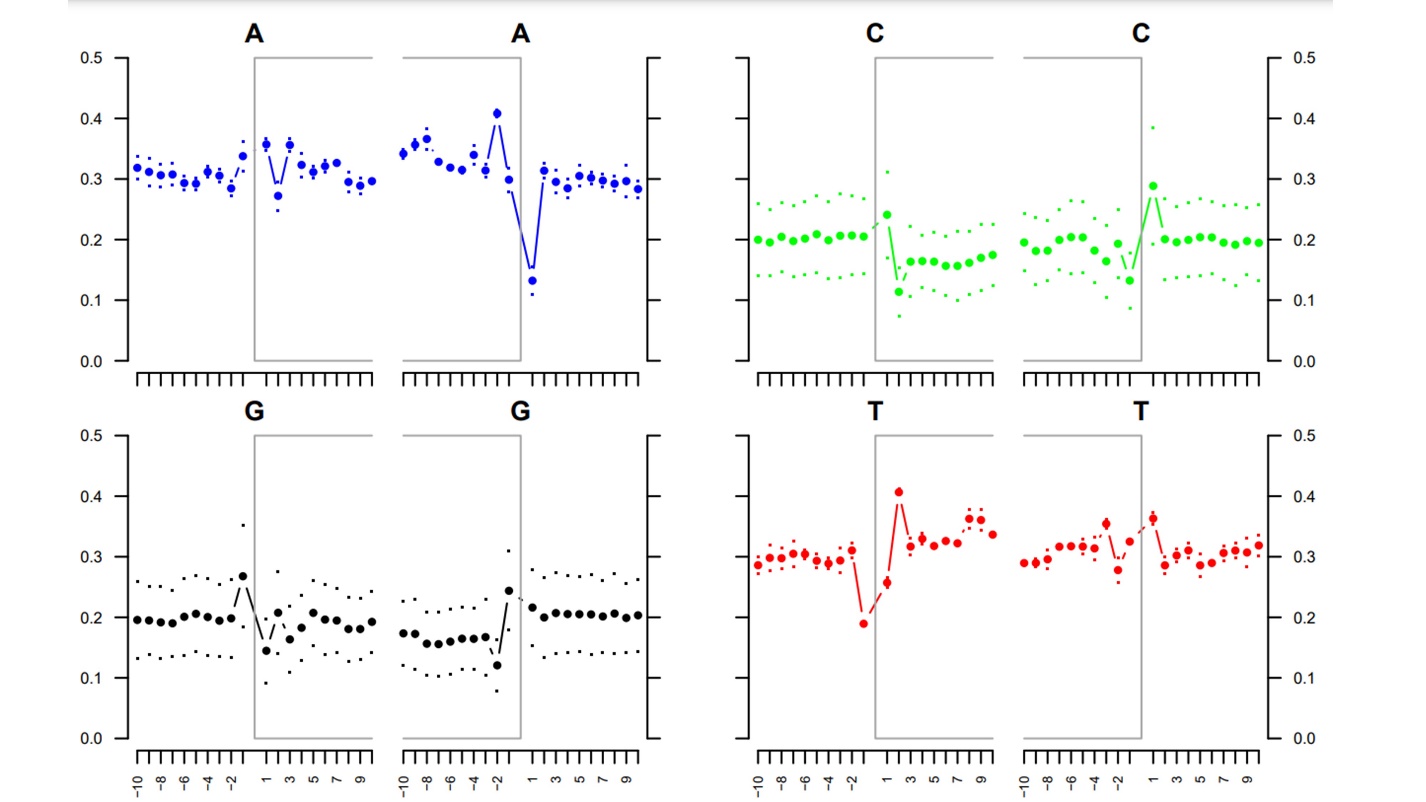


Supplemental Figure 4: mapDamage base frequencies for #73052.


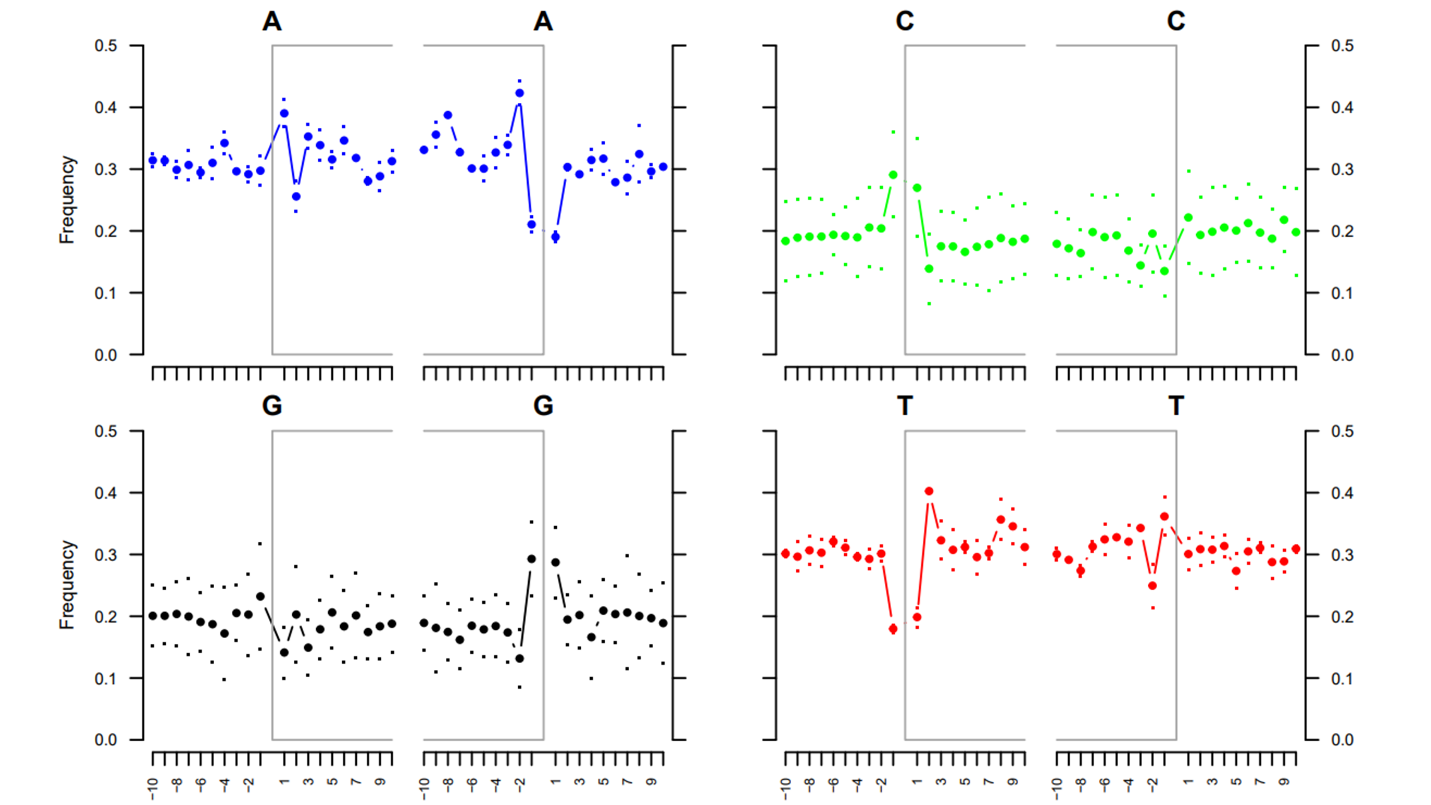


Supplemental Figure 5: mapDamage base frequencies for #75943.


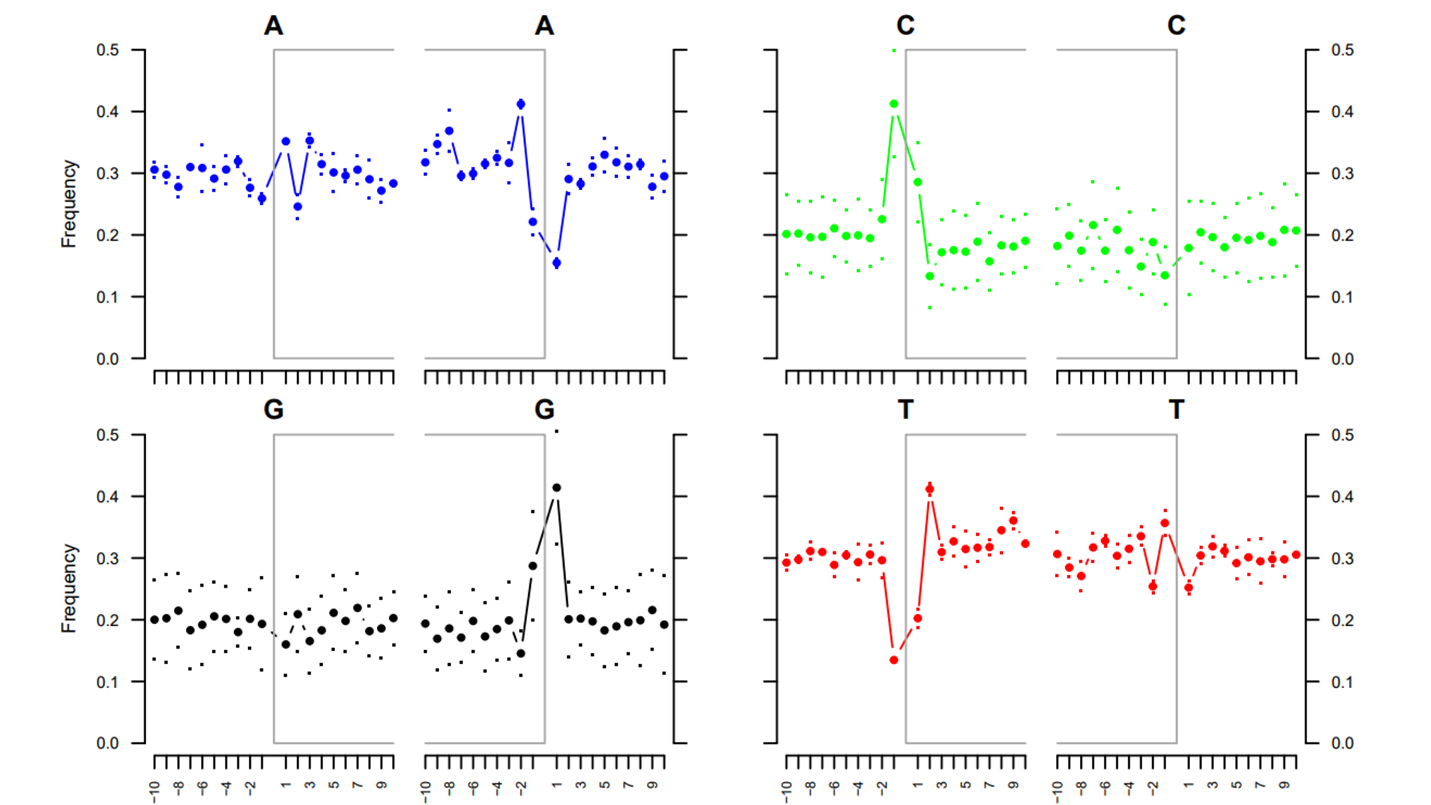


Supplemental Figure 6: mapDamage base frequencies for #135139.


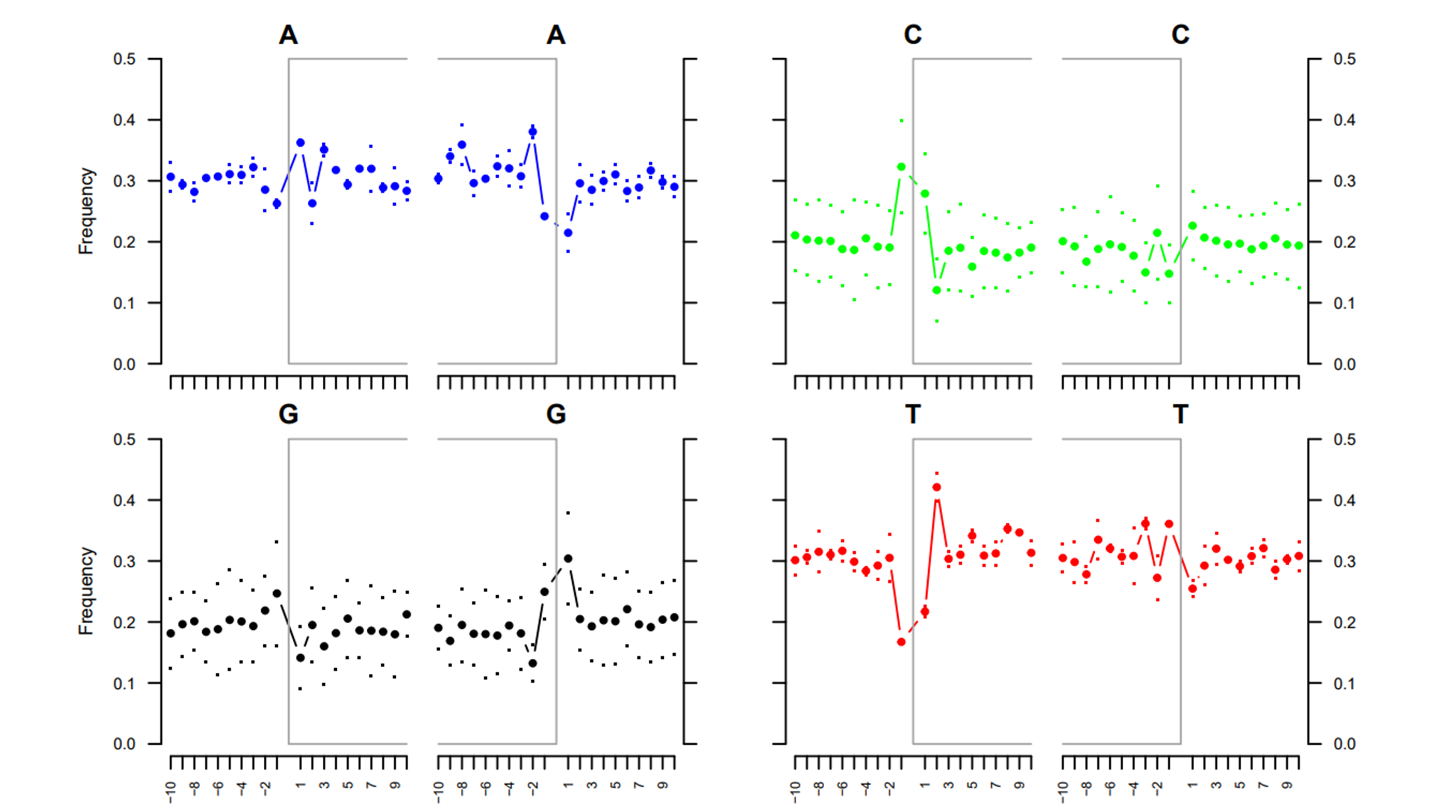


Supplemental Figure 7: Misincorporation for samples A) #118236 and B) #68100.


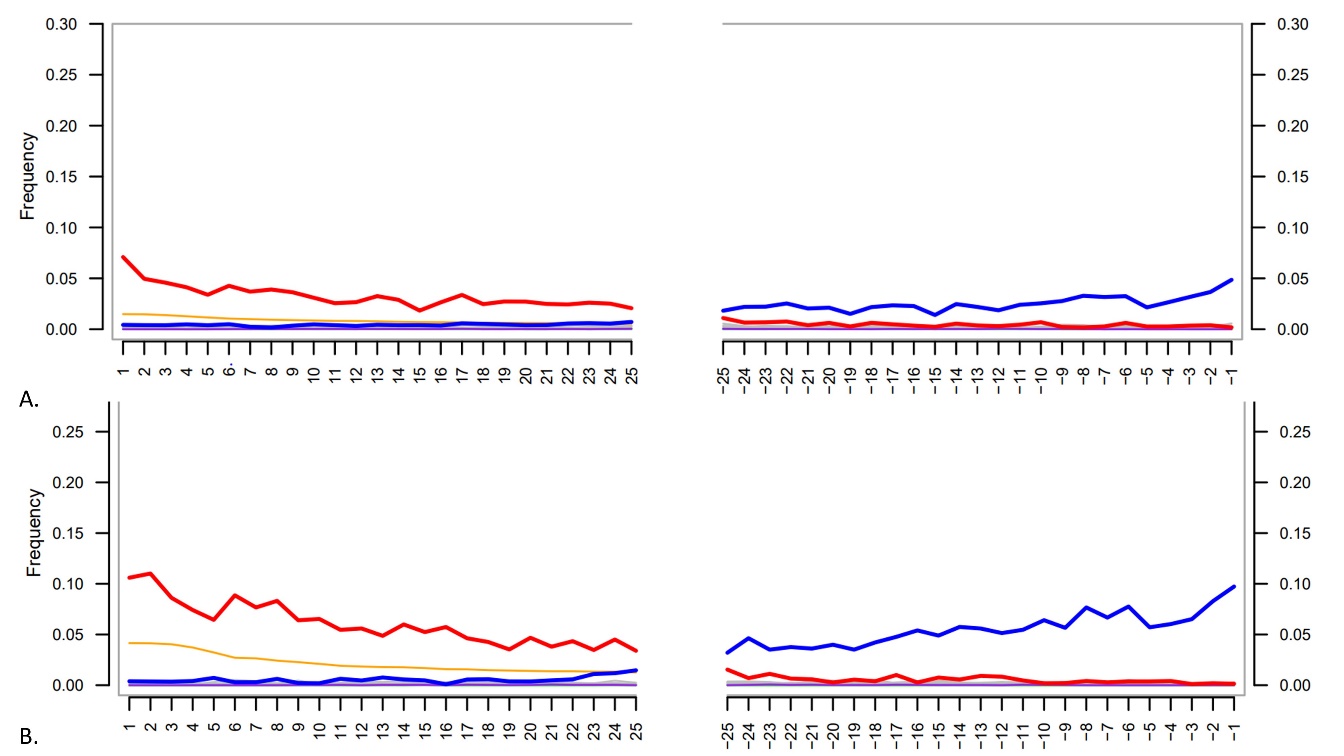


Supplemental Figure 8: Misincorporation for samples A) #118232 and B) #73052.


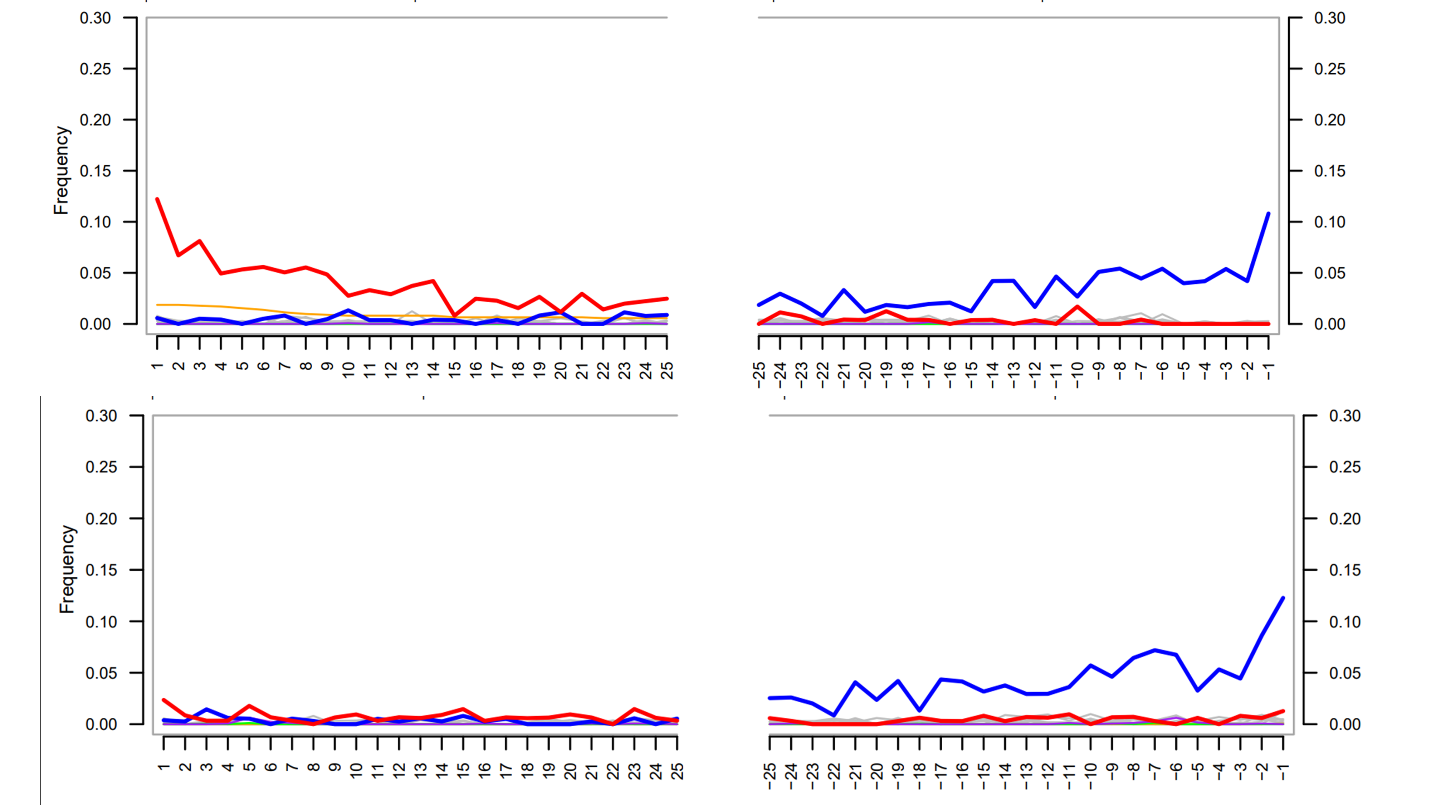


Supplemental Figure 9: Misincorporation for samples A) #75943 and B) #135139.


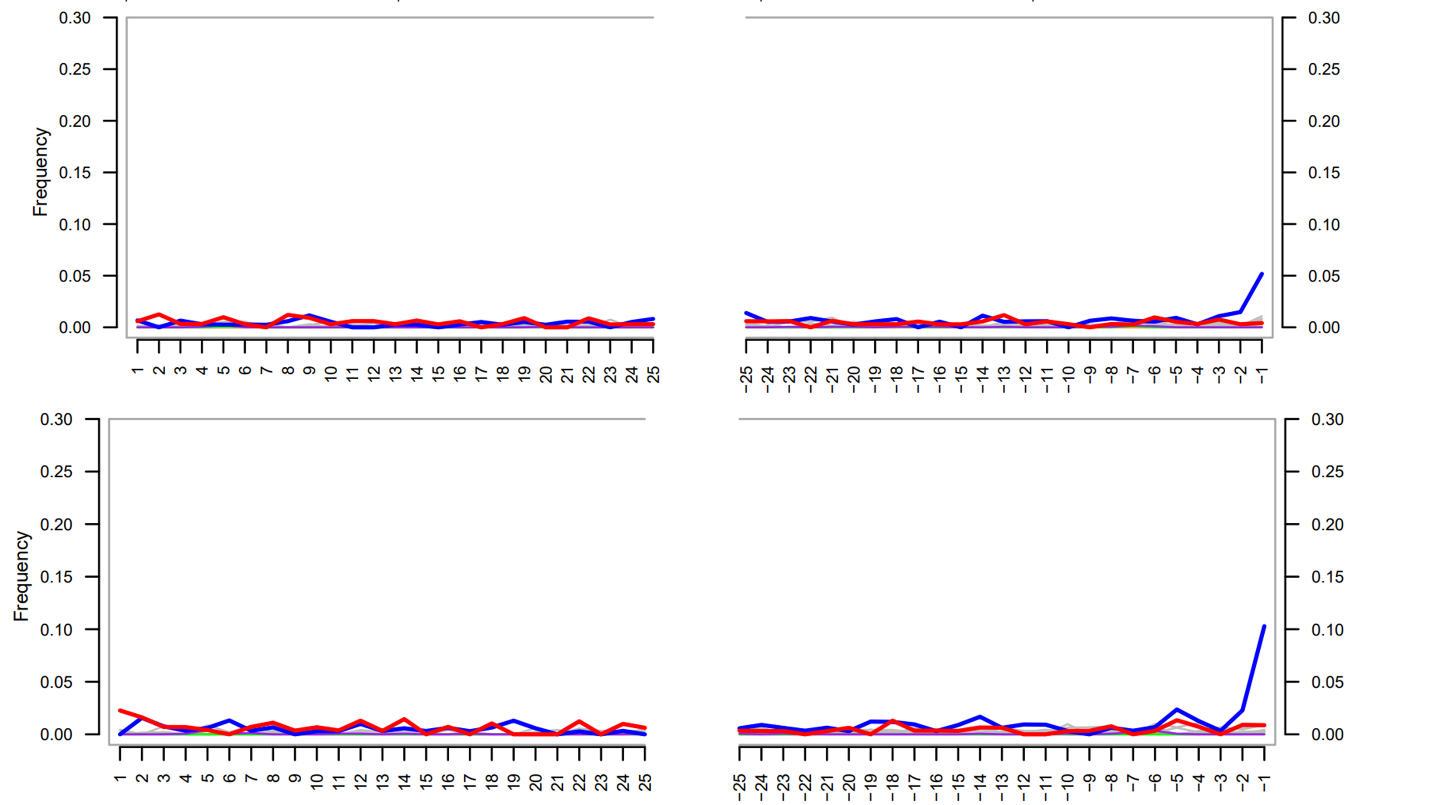

Supplement: Supplementary Text 4 — Supplemental Text 4. Sequence Authenticity. [file NIHMS2070856-supplement-Supplementary_Text_4.docx]
