# Supplementary material for: The Dogs of Tsenacomoco: Ancient DNA Reveals the Presence of Local Dogs at Jamestown Colony in the Early Seventeenth Century
Source: Am Antiq. Author manuscript; Available in PMC 2025 Aug 30. (PMC12395468; doi:10.1017/aaq.2024.25)
Supplement: Supplementary Text 1 — Supplemental Text 1. Archaeological Context for Jamestown Samples for More Detailed Descriptions of the Features. [file NIHMS2070856-supplement-Supplementary_Text_1.docx]

**Supplemental Text 1: Archaeological Context for Jamestown Samples**

Supplementary materials for:

The Dogs of *Tsenacomoco*: Ancient DNA Reveals Presence of Local Dogs at Jamestown Colony in Early Seventeenth Century

**Authors**

Ariane Thomas*

Matthew E. Hill, Jr.

Leah Stricker

Michael Lavin

David Givens

Alida de Flamingh

Kelsey E. Witt

Ripan S. Malhi

Andrew Kitchen

*****Corresponding author, [ariane-thomas@uiowa.edu](mailto:ariane-thomas@uiowa.edu)

^§^These authors co-supervised this work.

**Contents**

East Bulwark Trench (JR0082W Layer; Sample #114709)

Pit 5 (JR0731F and JR0731D Layers; Samples #22647 and #23799)

Pit 8 (JR1795D Layer; Samples #135142, #135143, and #135144)

Pit 9 (JR1530B Layer; Samples #52695, and #135140)

Early Well Deposits, Structure 185 (JR2718J, JR2718W and JR2718N Layers; Samples #118230 and #72592, #118236 and #7594, and #118231, #118232, #73052, #135786, and #74222)

Metalworking/Bakery Shop, Structure 183 (JR2361C Layer; Samples #135138 and #68100)

Pit 17 (JR2132H Layer; Sample #135777)

Second Well, Structure 177 (JR2158N Layer; Sample #135139)

Civil War Fort, Structure 145, (JR1892D Layer; Sample #118294)

Supplementary References Cited

**East Bulwark Trench (JR0082W Layer; Sample #114709)**

In 1996, James Fort’s eastern corner (bulwark) was discovered (Kelso and Straube 2004), one of the first significant features found by archaeologists which confirmed the rediscovery of James Fort. The bulwark trench was dug during the construction of the Fort as both a source of soil to strengthen the palisade, and as a defensive trench surrounding the fortified bulwark. Located ten feet outside the bulwark palisade and following along its curved line, numerous early Fort-period artifacts were found in the trench, which likely accumulated small amounts of trash during the Fort period and was filled in totally when Jamestown became a royal colony (AD 1624). Artifacts representing a military presence, including a complete fifteenth century doublet breastplate and elements of a buckler, a small leather covered shield, as well as artifacts representing the industrial nature of the colony, including glass and copper encrusted crucibles, vessels which withstand high-intensity heat, reinforced the hypothesis at the time that the original Jamestown fortification remained present on the landscape. In addition, domestic artifacts like iron bed bolts, seventeenth century ceramic vessels to contain shipped supplies like Midlands Purple butter pots, and faunal remains including both wild and domestic species speak to the permanent nature of the colony supplied by shipments from England. Sample #114709 was also excavated from the trench.

**Pit 5 (JR0731F and JR0731D Layers; Samples #22647 and #23799)**

An 8-foot-diameter and 3-foot-deep feature excavated in 2001 was identified as Pit 5. Little evidence remains of a structure overtop of the feature, but the rectangular-shaped bottom of the pit and artifacts recovered, including metal elements of a chest or casket, Delftware drug jars, iron needles, charred hickory nut fragments, and a Tsenacomocoan reed mat preserved by the presence of copper salts indicate the feature may have been an impermanent living space like Pits 8 and 9 (below). However, the location of the pit outside the walls of James Fort’s AD 1608 palisaded extension, and finds including over 50 glass beads, copper alloy rumbler bells, jettons, and copper scrap suggest Pit 5 may have been part of a trading area. Artifacts in the upper 7 of the 8 discrete layers in the feature indicate that it was filled with trash during the AD 1610 cleaning campaign which initiated the filling of numerous features on site with early Fort-period trash.

Pit 5 contained over 5,000 bones, with wild species including sturgeon, freshwater catfish, white perch, and gray squirrel dominating the assemblage. Faunal remains from crabs, opossums, chicken, duck, diamondback terrapins, bears, bobcats, and canids (#22647 and #23799) were also recovered. There is minimal weathering of faunal bones, but burning, carnivore gnaw marks, and butchery marks were present on both domestic and wild species recovered from Pit 5 (Andrews 2008).

**Pit 8 (JR1795D Layer; Samples #135142, #135143, and #135144)**

Interpreted as one of at least four early Fort-period impermanent storage or living spaces colloquially called “soldier's pits”, Pit 8, is situated as the furthest south in a series of pits along the interior of the west palisade wall of James Fort. The presence of early seventeenth century artifacts including armor elements, buckles, lead shot and shot making tools, as well as high status household and personal items including small decorative spangles, a religious medallion, Façon de Venise drinking glass fragments, and a bone chess piece indicate that the pit may have been associated a high-status individual with a military background (Kelso and Straube 2008; McKnight 2012). However, Pit 8 is notable for its dense concentration of tobacco pipe fragments, a smoker’s companion, and tobacco pipe production tools and waste products. A fragmentary, but highly significant vessel, made by pressing clay into an Indigenous basket before firing was also recovered from this feature (Straube 2011). These items are likely associated with Robert Cotton, a pipemaker, who arrived in Virginia in AD 1608, and represents an exchange of knowledge and goods perhaps between Cotton himself and Tsenacomocoan individuals.

Significant amounts of Hickory (*Carya sp*.) nut shells (McKnight 2012), and at least 23 freshwater catfish suggest that food preparation or consumption occurred in or near the pit. The faunal assemblage (bone count = 7,059) shows that Pit 8 contained Starving Time period trash, with butchery marks found on pig, cattle, deer, beaver, and dolphin bones. Similar to Pit 5, wild MNIs outnumber domesticated MNIs. Some bones were burned, others were gnawed by a rodent, and pig, cow, deer, and goose bones have carnivore puncture holes and other gnaw marks (Andrews 2008).

**Pit 9 (JR1530B Layer; Samples #52695, and #135140)**

Pit 9 is adjacent to Pit 8, the next in line in a series of pits along the interior of the west palisade wall of James Fort, possible impermanent storage or living spaces referred to as “Soldier’s Pits”. This pit measured 5’8”x4’6” and 1’5” deep, significantly smaller than Pit 8, and contained about half as many artifacts. Of those recovered, like Pit 8 they suggest a militarily affiliated individual of high status. Finds included Martincamp flask fragments, mail, firearm elements, Raeren Stoneware oil lamp fragments and a bone pen. Over 400 sherds of Native pottery were recovered, and crossmended English ceramics between Pits 8, 9, and 10 suggest that they were filled at the same time with similar trash (Kelso and Straube 2008). All three pits contained canid remains.

Pit 9 had fewer faunal bones than Pit 8, only about 3,225 (Andrews 2008). High frequencies of sturgeon, box turtle, gar, and catfish bones were found in this feature for both NISP and MNI counts (Andrews 2008). Deer, crab, box turtle, sturgeon, and four indeterminate remains had been burned (Andrews 2008). Carnivore gnaw marks were found on deer, cow, goose, and box turtle bones. Deer, cow, pig, and horse bones had butchery marks (Andrews 2008).

**Early Well Deposits, Structure 185 (JR2718J, JR2718W and JR2718N Layers; Samples #118230 and #72592, #118236 and #7594, and #118231, #118232, #73052, #135786, and #74222)**

Structure 185, located near the center of the triangular James Fort, was excavated in 2009 (Kelso and Straube 2012). The feature extended 14 feet below the ground surface and was likely constructed and used as a well in a cellar in AD 1608. The well was quickly abandoned because it could not provide a consistent source of fresh water. It was filled with trash in AD 1610, likely during a cleaning campaign Lord De La Warr initiated soon after his arrival. The fill represents various refuse layers, including over 500,000 artifacts, many deposited during the Starving Time winter of AD 1609 – 1610. Notable among the many artifacts are finds consistent with an early Fort-period date, including arms, armor, glass beads used in trade, late 16^th^ century and early 17^th^ century European ceramics, and thousands of oyster shells, in addition to well preserved organic material like European cloth fragments, and cask staves from the wooden cask placed at the bottom of the well by the colonists.

Over 10,000 sherds, representing at least 10 Native-made ceramic vessels were recovered from the First Well, far more than any other feature on the site. In addition, over 5,000 *Geukensia demissa* mussel shell beads, as well as *Geukensia demissa* shells and fragments were recovered from the feature.

The faunal assemblage from Structure 185 has not undergone formal identification and analysis, but preliminary sorting identified a variety of domesticated and wild faunal material, including sheep or goat, horse, turtle, squirrel, raccoon, woodchuck, snake, sturgeon, dolphin, blue crab, shark, and other fish, in addition to canid remains. Bones of the Cahow, a Bermudian petrol, and a green turtle carapace indicate that some material came from survivors of the Sea Venture shipwreck, who spent the winter of AD 1609 – 1610 in Bermuda. Initial investigations indicate that the faunal assemblage from this feature will align with findings from other Starving Time features, with wild species making up the majority of the assemblage, and domesticated species revealing patterns of supply from England.

Canid remains in this study were recovered from layer JR2718J (#118230 and #72592), likely one of the uppermost layers of an original backfilling effort, layer JR2718N (#118231, #118232, #73052, #135786, and #74222), the largest layer in the feature by volume, and JR2718W (#118236 and #75943), the first layer of deposition after the well was abandoned (Kelso and Straube 2012).

**Metalworking/Bakery Shop, Structure 183 (JR2361C Layer; Samples #135138 and #68100)**

Structure 183 is a cellar feature excavated during the summer of AD 2006 (Kelso and Straube 2012). The complexity of the feature suggests that Structure 183 was reused multiple times before its eventual abandonment. The cellar was likely part of a metalworking shop first used around 1608 AD. By June AD 1610, colonists transformed the cellar into a kitchen complete with the construction of two domed brick ovens. Eventually, the ovens fell into disrepair, and the structure was abandoned and filled with trash sometime during AD 1617 – 1624.

Layer JR2361C represents the top of the cellar fill that was deposited as late as AD 1617 during a campaign of new building construction within James Fort (Kelso and Straube 2012). The recovery of a Harrington Farthing coin dated AD 1613, provides a secure *terminus post quem* (earliest possible date) for layer JR2361C. Despite the post-AD 1613 deposition date for JR2361C, like the Second Well feature, crossmended ceramics, which include sherds from both Structure 183 and Structure 185 (in JR2718N, JR2718W, and JR2361C) indicate that trash dating to the Starving Time was mixed with later material and deposited in the upper layers on Structure 183. Thus, some canid remains found in JR2361C may date as early as the Starving Time winter of AD 1609 – 1610. Formal zooarchaeological analysis and identification has not yet occurred for Structure 183, however some select species have been preliminarily identified. This included two fragments of a left maxilla identified as *Canis* recovered from this stratum. A left maxillary fragment with premolar (second, third, fourth) and molar (first) teeth was labeled #68100.

**Pit 17 (JR2132H Layer; Sample #135777)**

The function of the Pit 17, which measured 8'4" x 5'2" and 2'5" deep is unknown. Based on its relationship to nearby features, the pit was likely filled prior to AD 1611. Pit 17 was filled with heavy concentrations of clinker and charcoal, waste from iron working (Kelso *et al.* 2012), which may have come from the nearby Metalworking Shop (Structure 183) as it was being converted into a bakery around the same time. Other artifacts, including Chinese porcelain vessels, Nueva Cadiz glass beads, and case bottle glass support an early Fort-period date (Kelso and Straube 2008). Layer JR2132H, the layer from which canid sample #137777 was excavated, is in the center of the feature and contained several iron dagger pommels, copper alloy bandoliers, and iron scissors (Kelso and Straube 2008). Over 450 sherds of Indigenous-made pottery were present in the feature, as were 18 *Geukensia demissa* shell beads. Additionally, a few fired clay balls were recovered from the pit, which may have been used to cook or keep food warm, were part of a game, or were accidentally fired measures of clay for tobacco pipe production, perhaps occurring in nearby Pit 8.

**Second Well, Structure 177 (JR2158N Layer; Sample #135139)**

James Fort residents dug the Second Well AD 1610 – 1611, and it was filled with refuse, including sample #135139, by AD 1617 – 1618 (Bowen *et al.* 2021, Kelso and Straube 2012). The well was 16 feet deep and contained approximately 200,000 artifacts. Among these were extremely well-preserved organic material including about 10 leather shoes, a wooden bowl, over 4,000 hickory and black walnut nutshells, and tobacco seeds. An intact and loaded Roman-lock pistol and a polearm weapon called a halberd that included heraldic devices of the De La Warr coat of arms were also recovered from the feature, speaking to the continued military nature of the fortification, and the social status of some of the residents. Despite the post-Starving Time date of the features fill, crossmended ceramics between this feature and the first well suggest that Starving Time period trash was used to fill both wells after they no longer provided potable water.

The Second Well contained over 170,000 faunal remains from 13 layers of fill. Recent analysis identified five elements from layers JR2158 H, P, and U as *Canis lupus familiaris* and another five bones as *Canis*, indicating the possibility of wolf remains in layer JR2158N. No *Canis* remains in layer JR2158N had scorch marks or butchery marks, while other species (swine, deer, and cattle) did. Of the overall faunal assemblage, element distribution patterns, kill-off data, and biomass results for livestock revealed the importance of swine over cattle, the protection of herds to promote stability and growth, and the significance of imported provisions. Large quantities of wild mammals, fowl, and fish indicate that the colonists utilized their environment to supplement their diet. Faunal analysis of the second well elucidated Jamestown’s development from dependence on the Virginia Company and Virginia Indians for subsistence into a stable colony, moving towards self-sufficiency (Bowen *et al.* 2021).

**Civil War Fort, Structure 145, (JR1892D Layer; Sample #118294)**

One sample, #118294, came from layer JR1892D of Structure 145. Structure 145 is an earthwork fortification known as Fort Pocahontas, constructed by Confederate troops during the Civil War (AD 1861), and partially covers the previous location of the earlier James Fort. No battles were fought on Jamestown Island during the Civil War and after Fort Pocahontas was abandoned by the South in AD 1862, Union troops and freed slaves inhabited Jamestown Island for the duration of the War. In addition to the Fort itself, which contained two expense magazines (Structures 169 and 182) and a bombproof structure (Structure 171), a surrounding dry moat and a Civil-War-period roadway with ditches on either side have also been located and archaeologically investigated on the site. The construction of the Civil War Fort and other associated features disrupted seventeenth century stratigraphic layers and features, and numerous early seventeenth artifacts were displaced in the mounded earth.

**Supplementary References Cited**

Andrews, Susan Trevarthen.

2008 Faunal Analysis of James Fort: Structure 166, Pit 5, Pit 8, Pit 9, Pit 10, and Pit 11. Report to the Jamestown Rediscovery Project, Jamestown, VA.

Bowen, Joanne, Susan Trevarthen Andrews, and Stephen C. Atkins.

2021 *Jamestown Colony: From Food Dependence to Food Independence. Faunal Analysis for Second Well (JR2158): Layers H, N, P, U, X, and AA.* Field Reports. Preservation Virginia.

Kelso, William M., and Beverly Straube

2012 2007-2010 Interim Report on the Preservation Virginia Excavations at Jamestown. Field Reports. Preservation Virginia.

McKnight, Justine W.

2012 Archeobotanical Analysis of Feature Fill from Three James Fort Period Features: A Pilot Study. Report to the Jamestown Rediscovery Project, Jamestown, VA.

Straube, Beverly A.

2011 Incorporating the Other: A Seventeenth-Century Virginia Indian Basket Pot Rendered in Clay. Ceramics in America.
