# Supplementary material for: The Dogs of Tsenacomoco: Ancient DNA Reveals the Presence of Local Dogs at Jamestown Colony in the Early Seventeenth Century
Source: Am Antiq. Author manuscript; Available in PMC 2025 Aug 30. (PMC12395468; doi:10.1017/aaq.2024.25)
Supplement: Supplementary Text 2 — Supplemental Text 2. Low-Quality Data for Fourteen Jamestown Canids. [file NIHMS2070856-supplement-Supplementary_Text_2.docx]

**Supplemental Text 2: Low-quality Data for Fourteen Jamestown Canids**

Supplemental materials for:

The Dogs of *Tsenacomoco*: Ancient DNA Reveals Presence of Local Dogs at Jamestown Colony in Early Seventeenth Century

**Authors**

Ariane Thomas*

Matthew E. Hill, Jr.

Leah Stricker

Michael Lavin

David Givens

Alida de Flamingh

Kelsey E. Witt

Ripan S. Malhi

Andrew Kitchen

*****Corresponding author, [ariane-thomas@uiowa.edu](mailto:ariane-thomas@uiowa.edu)

^§^These authors co-supervised this work.

**Contents**

Description of Methods

[Supplemental Figure 1: Majority rule maximum likelihood tree of ancient and modern canids and low-quality Jamestown sequences to estimate species ancestry of the mitogenomes (n = 905). Vulpes vulpes was used as an outgroup and is marked in blue. Ancient and modern wolves are colored black, modern coyotes are green, dingoes are dark yellow, ancient dogs from Asia and Europe are purple, ancient dogs from the Americas are tan, and modern dogs are gray. The tree was constructed using the same specifications as the ML tree (Figure 4 in the main manuscript) except this ML tree was run with 100 bootstraps.](#_Toc163490821)

[Supplemental Figure 2: Magnification of the ancient North American clade in the maximum likelihood tree with low-quality Jamestown sequences to estimate species ancestry of the mitogenome. Jamestown samples are in red, Mesoamerican dogs are in a blue collapsed clade, and Siberian dogs are in a green collapsed clade.](#_Toc163490822)

[Supplemental Figure 3: Magnification of clade containing #135138, #23799, and #74222, in red, in the maximum likelihood tree to estimate species ancestry of the mitogenome.](#_Toc163490823)

[Supplemental Figure 4: Magnification of clade containing #22647, in red, in the maximum likelihood tree to estimate species ancestry of the mitogenome.](#_Toc163490824)

[Supplemental Figure 5: Magnification of clade containing #135786 and #135144, in red, in the maximum likelihood tree to estimate species ancestry of the mitogenome.](#_Toc163490825)

[Supplemental Figure 6: Magnification of clade containing #114709, in red, in the maximum likelihood tree to estimate species ancestry of the mitogenome.](#_Toc163490826)

Supplemental Table 1: Species Estimation of Low-Quality Jamestown Genomes.

**Description of Methods**

To estimate the species of low-quality samples from Jamestown, we used the same program, PALEOMIX v1.3.7 to map the reads to reference. ANGSD parameters were adjusted to increase the number of bases. The mapping quality score equal to or greater than 25 (-minMapQ 25) and minimum depth at 2 (-setMinDepth 2).

We input each consensus sequence into NCBI Basic Local Alignment Search Tool (BLAST) and recorded the frequency of the top 100 hits to *Canis lupus*, *Canis lupus familiaris*, dingoes, and *Canis latrans*, shown in Supplemental Table 1. The frequency range for wolf ancestry was 0-76% with the majority of sequence hits to wolf below 7%. Only three samples #23799, #135143, and #135777 had higher hits to wolf (76%, 73%, 22%, respectively). Three had 100% of the hits to dog, #135138, #74222, and #118236. There was only one hit to dingo, #118232, and one hit to coyote, #135140.

BLAST has a variety of useful functions, but it also has limitations. BLAST can be used to identify species, generate a phylogenetic tree of results, and can map DNA to its location within the genome. In this study, we used it for species identification of the entire mitochondrial genome. However, BLAST uses a heuristic to find short matches between two sequences which it uses to start a local alignment. This means that BLAST does not always find the optimal alignment among the tens of thousands of canid sequences available in its database. For this reason, we aligned the fourteen low quality Jamestown sequences to the large sample of ancient and modern dogs, wolves, coyotes, dingoes, and foxes, shown in Supplemental Figure 1. We also magnified the clades of each of the Jamestown canid to view their closest relatives (Supplemental Figures 2-6). All of the canid samples grouped with modern or ancient dogs, seven of which grouped in the ancient North American dog clade.

Supplemental Figure 1: Majority rule maximum likelihood tree of ancient and modern canids and low-quality Jamestown sequences to estimate species ancestry of the mitogenomes (n = 905). Vulpes vulpes was used as an outgroup and is marked in blue. Ancient and modern wolves are colored black, modern coyotes are green, dingoes are dark yellow, ancient dogs from Asia and Europe are purple, ancient dogs from the Americas are tan, and modern dogs are gray. The tree was constructed using the same specifications as the ML tree (Figure 4 in the main manuscript) except this ML tree was run with 100 bootstraps.


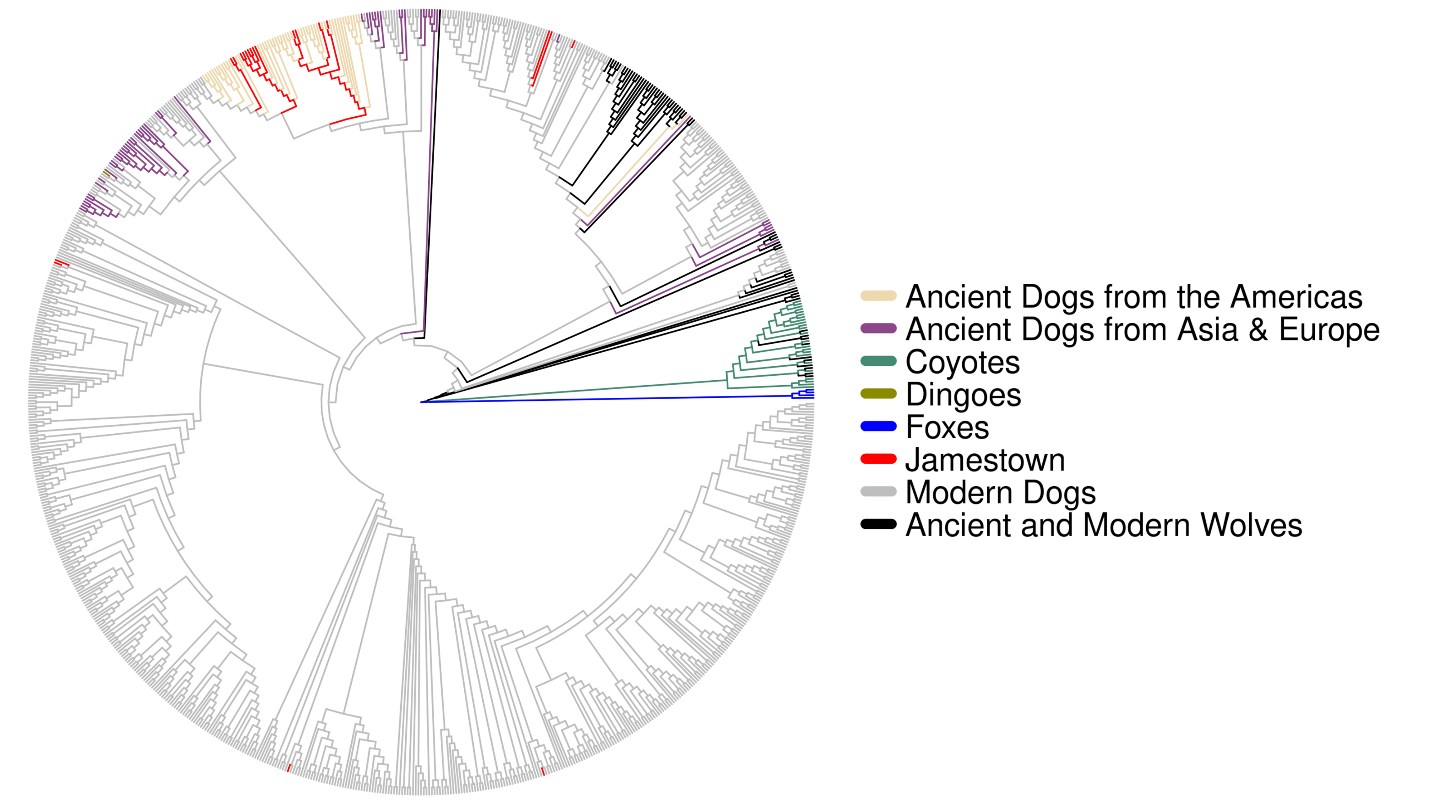


Supplemental Figure 2: Magnification of the ancient North American clade in the maximum likelihood tree with low-quality Jamestown sequences to estimate species ancestry of the mitogenome. Jamestown samples are in red, Mesoamerican dogs are in a blue collapsed clade, and Siberian dogs are in a green collapsed clade.


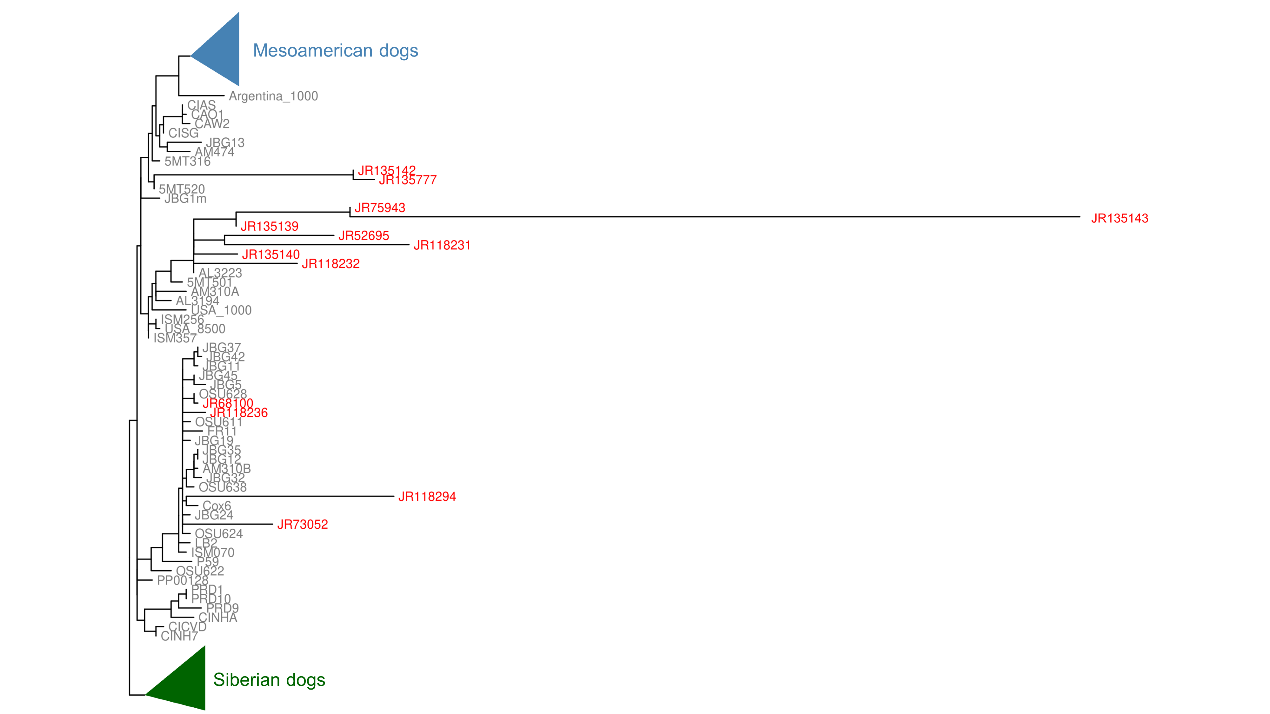


Supplemental Figure 3: Magnification of clade containing #135138, #23799, and #74222, in red, in the maximum likelihood tree to estimate species ancestry of the mitogenome.


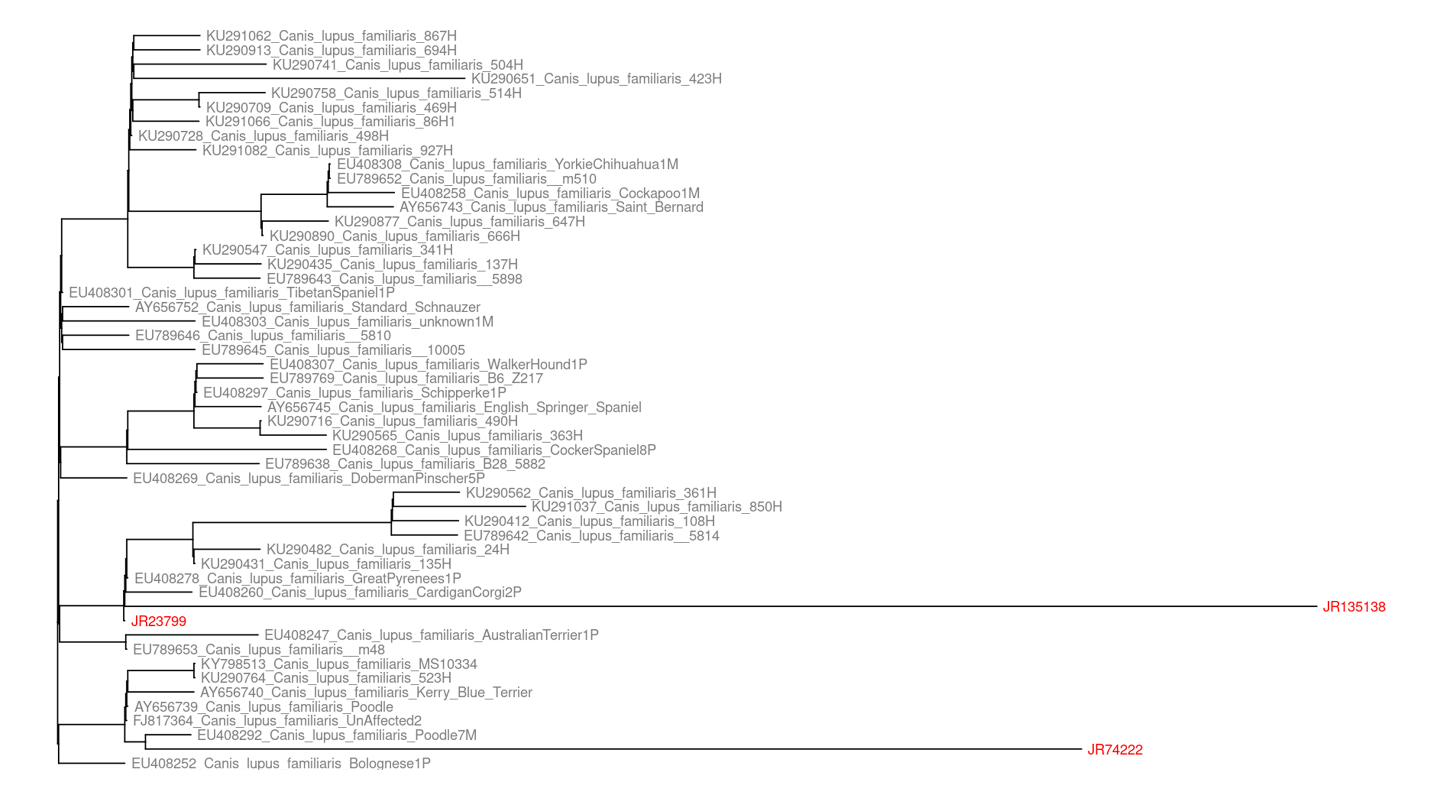


Supplemental Figure 4: Magnification of clade containing #22647, in red, in the maximum likelihood tree to estimate species ancestry of the mitogenome.


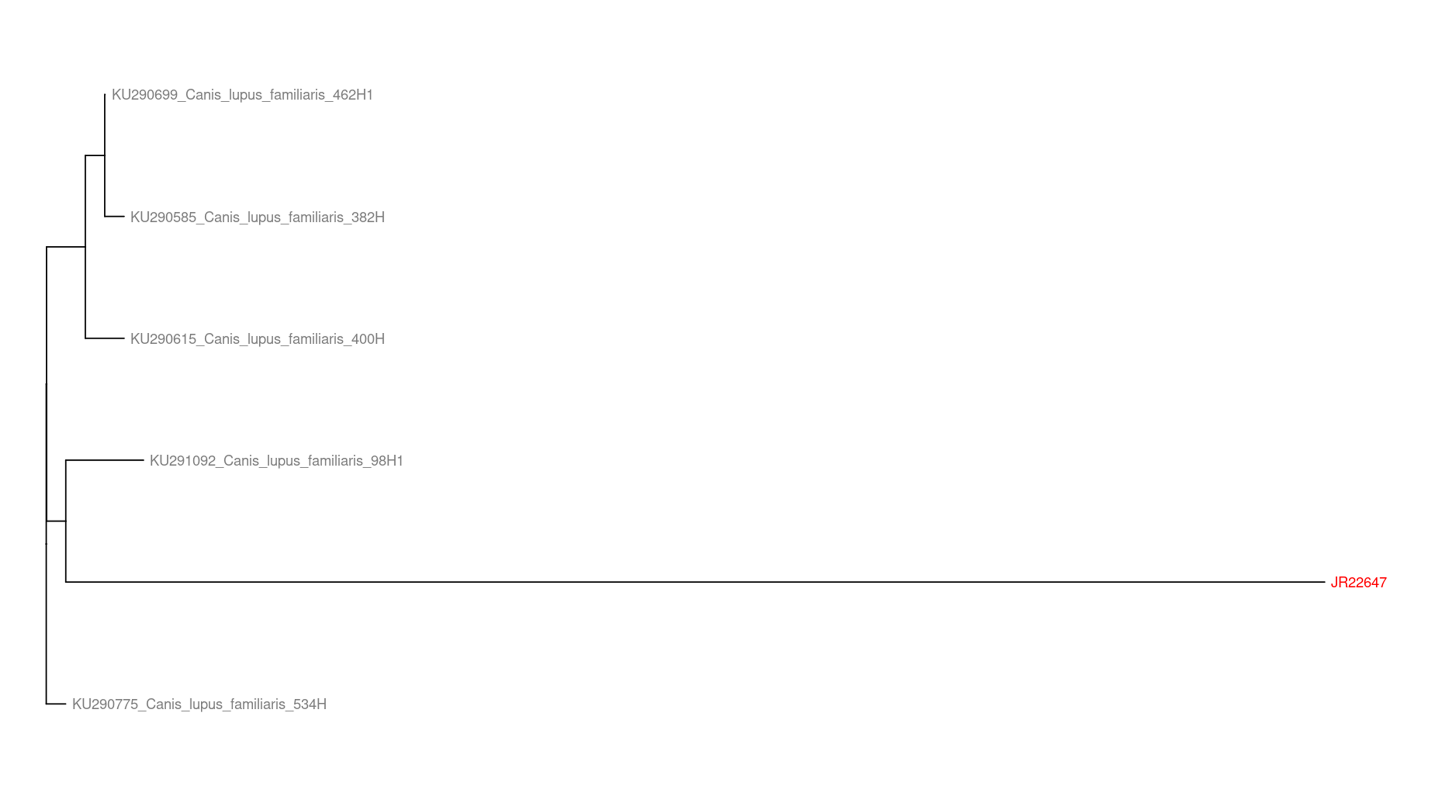


Supplemental Figure 5: Magnification of clade containing #135786 and #135144, in red, in the maximum likelihood tree to estimate species ancestry of the mitogenome.


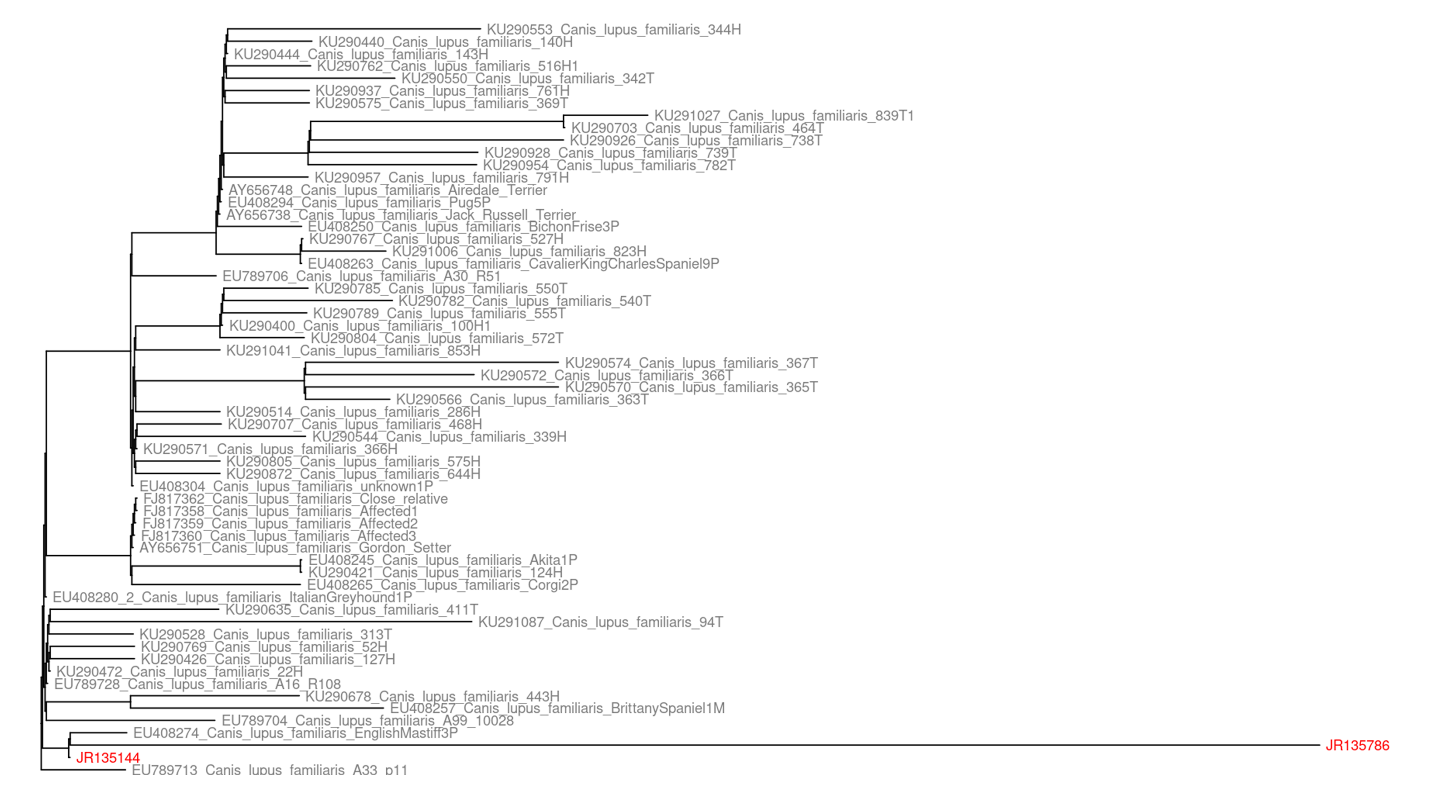


Supplemental Figure 6: Magnification of clade containing #114709, in red, in the maximum likelihood tree to estimate species ancestry of the mitogenome.


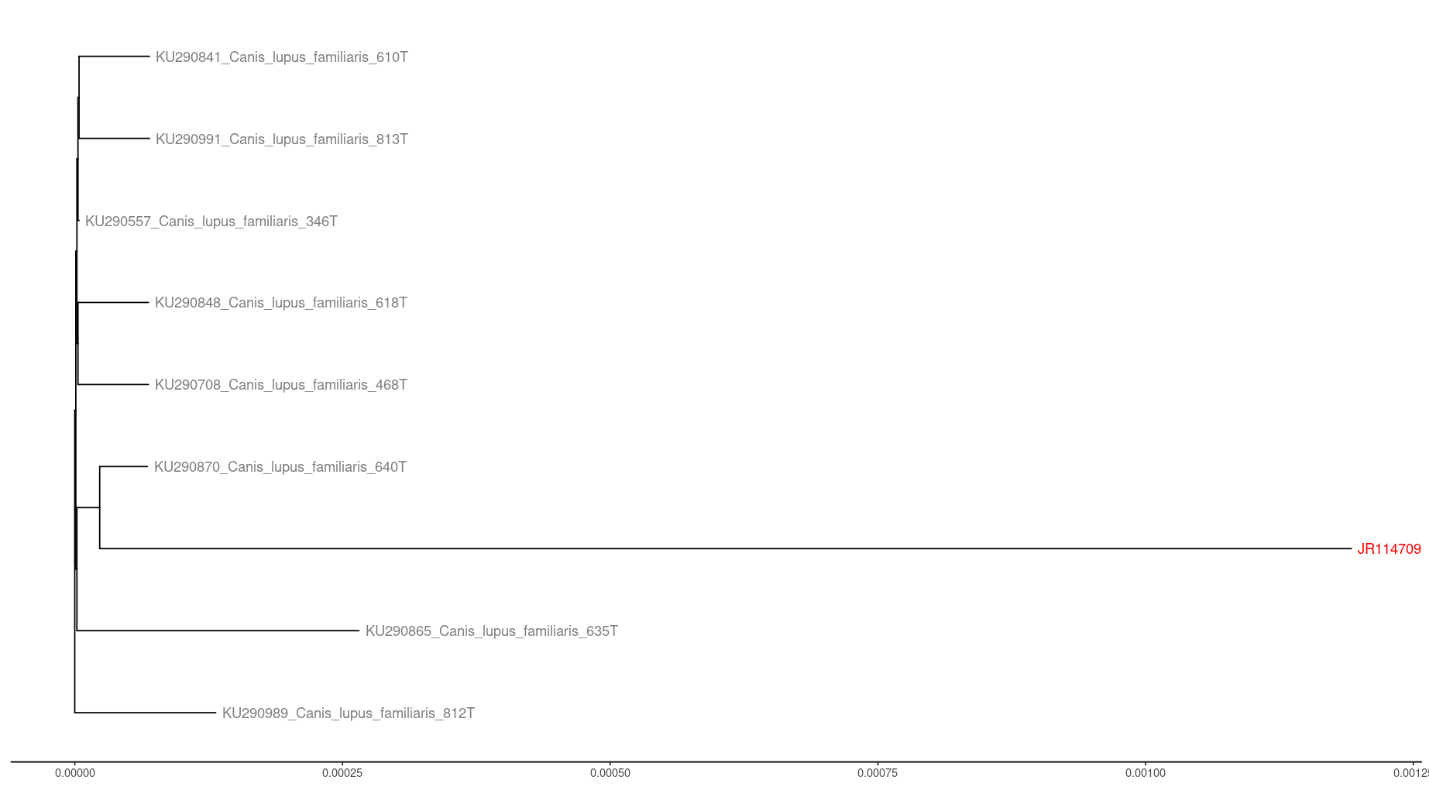


Supplemental Table 1: Species Estimation of Low-Quality Jamestown Genomes.

| Sample ID | mtDNA Coverage | BLAST Top 100 Hits | Genetic Affiliation of Nearest Sample in ML Tree |
| --- | --- | --- | --- |
| JR_23799 | 0.185986728 | 76/100 wolf, 24/100 dog | Modern dog |
| JR_114709 | 0.273808812 | 2/100 wolf, 98/100 dog | Modern dog |
| JR_135143 | 0.427273271 | 73/100 wolf, 27/100 dog | ancient North American dog |
| JR_135142 | 0.668260896 | 3/100 wolf, 97/100 dog | ancient North American dog |
| JR_57981_135777 | 0.679081724 | 22/100 wolf, 78/100 dog | ancient North American dog |
| JR_135140 | 0.78011598 | 1/100 wolf, 1/100 coyote, 98/100 dog | ancient North American dog |
| JR_135138 | 0.965445089 | 100/100 dog | Modern dog |
| JR_22647 | 1.040593053 | 3/100 wolf, 97/100 dog | Modern dog |
| JR_135144 | 1.157768877 | 5/100 wolf, 95/100 dog | Modern dog |
| JR_52695 | 1.185568243 | 4/100, 96/100 dog | ancient North American dog |
| JR_118231 | 1.342201232 | 3/100 wolf, 97/100 dog | ancient North American dog |
| JR_135786 | 1.539427273 | 5/100 wolf, 95/100 dog | Modern dog |
| JR_74222 | 2.590721588 | 100/100 dog | Modern dog |
| JR_118294 | 4.140730555 | 4/100 wolf, 96/100 dog | ancient North American dog |
| JR_118232 | 5.318287798 | 4/100 wolf, 1/100 dingo, 95/100 dog | ancient North American dog |
| JR_135139 | 9.146589347 | 3/100 wolf, 97/100 dog | ancient North American dog |
| JR_73052 | 9.56973755 | 5/100 wolf, 95/100 dog | ancient North American dog |
| JR_75943 | 10.54331321 | 7/100 wolf, 93/100 dog | ancient North American dog |
| JR_68100 | 25.38088121 | 3/100 wolf, 97/100 dog | ancient North American dog |
| JR_118236 | 66.78507802 | 100/100 dog | ancient North American dog |
